# Spirit of the Foreign Periodicals, &c.

**Published:** 1844-04-01

**Authors:** 


### Spirit of the Foreign Periodicals.

### M. Andral on Pathological Hematology.

Hematology, or that branch of medical science which is devoted to the study of the blood in the conditions of health and in disease, is by no means a creation of the present age. It is exactly a century ago since *Thomas Schwenke* published, under the very appellation now used, a treatise on this subject. M. *Andral,* with all the dignified impartiality which has ever characterised his conduct as a scientific man, has, at the commencement of his recent Essay, pointed out the most important labours of his predecessors in this interesting department of physiological research, and at the same time, he distinctly disclaims for himself all merit as to either the originality or the completeness of his present enquiries.

The time is not far distant when the question as to the alterations of the Blood was almost entirely overlooked by medical writers, or at most was alluded to only in a casual and very superficial manner. One party in medical literature, having their attention absorbed with the speculations of a mysterious Vitalism, were willing to accord only a very subordinate importance to all organic lesions, whether of the fluids or of the solids of the body; and thus it was that, in general, they became the more prolific of theoretical explanations of disease just in proportion as their ignorance of facts was the greatest. Another party erred quite as much by going an extravagant length in the very opposite direction; viz. by attaching an undue importance to every appreciable (however faintly this might be) lesion or alteration of structure, to be found on post-mortem examinations. Both these parties or schools regarded with equal indifference and neglect the problems of a Humoral Pathology. Even the very appearance of the blood, drawn in venæsection, was seldom noticed with any degree of precision; it was rather the result of a mere professional habit, than with any definite object of enquiry, that the buffy condition of the blood drawn was observed and recorded; and, although some of the terms of the old Humoral school were still retained in medical language, its doctrines and tenets were never mentioned but with ridicule and contempt. Many of our readers will perhaps be led, by these passing remarks, to recal to their minds the shadows of their early studies, and the lineaments of their first preceptors.

The absolute necessity of bringing to the very foremost ground, in all pathological enquiries, the exploration of the Solids of the body, of the Fluids which penetrate and permeate these, and of the vital forces to which both are alike subjected, has been insisted upon from the most remote antiquity; and medical men are now beginning to perceive that it is only upon the triple foundation of an exact and discriminating Solidism, Humorism, and Vitalism, that any truly sound system of pathology can ever be established. The crying sin of the ancient Humoral Doctrine consisted rather in its systematic usurpation of the totality of a science, one part only of which it could justly claim, than in the fanciful and erroneous hypotheses of which it was the parent source. The aim and object of the pathologist in these days seemed to have been, not so much the discovery of the various alterations induced by disease in the physical and chemical characters of the Fluids, as a fanciful explanation of the hidden mechanism of living bodies, and of the etiology of disease—not unfrequently by means of very forced analogies and suppositious resemblances.

The most ancient doctrine was, that the human body is composed of a few elementary or simple constituents, the abnormal conditions and irregular combinations of which formed, as it were, the key of every system of Pathogeny. This was the general view of medical science for many centuries. There was a difference of opinion, indeed, in different ages—according to the partial discoveries of a rudimentary chemistry—as to the nature of the elements in question; but the doctrine in all was essentially and fundamentally the same. The four elements of the Galenic pathology were, in course of time, replaced by the three—mercury, sulphur, and salt—of the Paracelsian; but, in either case, the foundation of the etiological reasoning was of the same erroneous character.

For several centuries, the advances and discoveries of chemistry served only to give rise to new and fanciful theories, and vague generalisations of disease. Acidity and alkalinity of the fluids were long the *Shibboleth* and watchwords of the schools; and, although these terms have been long banished from medical literature, there seems to be a manifest tendency, in the present age, to a re-adoption (in a modified form indeed) of the dogmas which they serve to designate. It is certainly curious to observe, that almost every medico-chemical theory, which has at any time been advanced, is found on examination to contain a certain *modicum* of truth—often blended indeed with no inconsiderable portion of error;—and also, that very rarely has any such theory been propounded, without encountering more or less opposition. *Robert Boyle* was a strenuous opponent of the medico-chemical doctrines of his day; *Sydenham* expressly protested against the 'new inventions of the chemists;' and later still, *Bordeu* made a very vigorous and unsparing assault upon what he deemed the errors of his cotemporaries in this department of pathological research. Taking a position midway between the extremes either way—of excessive admiration on the one hand, and undue disparagement on the other—*Haller* was led to enunciate that truly philosophic remark, which M. *Andral* has selected for the motto of his brochure:—*Non ideo analyses sanguinis utilitate suâ destituuntur, dum sapienter noverimus spes nostras recidere, neque plura nocere quam a naturâ discimus.*

The Microscope was not more fortunate in its results at first than the instruments of Chemistry; for the early discoveries, effected by its *means*, seemed only to open a new field to fanciful speculation. The presence of globules in the blood being once distinctly ascertained by its aid, there arose immediately the pathological sect—of which *Boerhaave* was the head—that endeavoured to found an etiological creed upon certain assumed data respecting the relative size of the blood-globules, and the calibre of the capillary vessels.

M. *Andral,* carefully avoiding the errors of his predecessors, alike in the chemical and in the microscopic fields of pathogenic enquiry, has applied his enlightened mind to the investigation of the more obvious changes, which the blood is found to exhibit in the course of many classes of disease. He has pointed out with great judgment the numerous sources of fallacy which are apt to mislead the inexperienced in their hematological researches. We may mention one example.

It has been too commonly imagined that the blood of different tribes of animals presents nearly the same characters. This is not so. What is normal in one species is a sign and token of morbid alteration in another. The buffy coat very generally indicates the existence of inflammation in the blood of man; whereas, with a little management on the part of the veterinary phlebotomist, this phenomenon may be made to appear at any time on the blood of the horse. In the same animal species, the different elements of the blood may vary a good deal within certain limits, without there being of a necessity any diseased action present in the system at the time; and this too, in consequence not only of peculiarities of individual organisation, but also of the kind of food on which the animal has been fed, as well as the treatment to which it has been previously subjected, and so-forth.

We need scarcely say that it is most necessary to be perfectly well acquainted with the physical characters of the blood in a state of health, before any person can undertake to examine with advantage the changes to which this fluid is liable during the progress of diseased action. Numerous mistakes have been committed from ignorance of the normal characters. The globules of a *framboisé* aspect have, on more than one occasion, been mistaken for globules altered by disease, or in the course of gradual destruction. M. *Andral* has shewn that the said globules are produced by the precipitation of white corpuscles around others of a red colour; and the researches, which have led him to adopt this conclusion, tend to evolve a general principle, that the blood, if examined just as it flows from a divided vessel, exhibits at first insulated white corpuscles, and afterwards a number of red globules which have an appearance that he describes as *framboisé et festonné;* but that, if it is deprived of all its fibrine before its spontaneous coagulation, it ceases to present this phenomenon under the microscope. From this circumstance, therefore, we may infer that the presence of fibrine is necessary for the development of white corpuscles in a drop of blood.

M. *Andral* arranges, under three heads, the abnormal changes of the blood that are discoverable by the microscope and chemical analysis. Under the *first* he describes those changes which consist in an alteration of the relative proportions of the component parts of the blood; the *second* comprises the modifications of these component parts in respect of their qualities and physical properties; and the *third,* those that are referrible to the formation of new principles which are not present in the normal condition of the fluid. It is the first only of these three categories that M. *Andral* has hitherto been able to investigate with any degree of completeness; and he is still occupied with prosecuting his enquiries on this subject, respecting the changes in the relative proportions of the globules, fibrine, and the solid contents of the serum. We cannot indeed praise too highly the sage caution evinced by our distinguished countryman in publishing his observations, before they have been well elaborated and satisfactorily established. There are so many ardent spirits now a days ready to produce complete treatises (!) on subjects which they have scarcely done more than skim, that we cannot but deem it fortunate for science when a high authority, like M. *Andral,* sets so conspicuous an example of patient reserve and laborious accuracy.

In a series of most interesting chapters, we have presented before us a description of the changes of the blood in the Pyrexiæ and the Phlegmasiæ, in Anemia, in Dropsy, in Hæmorrhages, and in the various Neuroses, as well as in a good many Organic Diseases. Already most of the facts, consigned in the pages of this truly important essay, are recognised as established truths in medicine, and circulate as axioms of classic truth in the doctrine of the schools. Within the course of a few months they have received universal verification; and no one can dispute that they have contributed in a very extraordinary degree, to popularise the examination of the blood, and to regulate the results which this means of analysis is calculated to produce in clinical experience.

In this respect, M. *Andral* has not only opened up a field of most curious research, and established the intimate analogy that may be traced between the data furnished by chemical and microscopic examination on the one hand, and those of clinical experience on the other; but he has also exercised a salutary influence on general therapeutics and practical medicine.

At the commencement of every disease, the blood may exhibit one of two modifications in the condition of its globules; these may be either in abnormal excess or deficiency. The one state belongs to Plethora, and the other to Anæmia: in the former, there is a *plus,* while in the latter there is a *minus,* in the relative proportion of this important element of the circulating fluid. These few words contain in substance the initial rule of all sound practice;—not that M. *Andral* has the merit of its discovery; for all that he has done is simply to have *proved* by a series of incontrovertible and indisputable facts what has been often said as to the force of constitution in different patients, under the operation of the same disease. To replace a *banality* in medical enquiries by a precise and positive formula, is to confer a service not only on science, but also on suffering humanity.

Does not the mere enunciation of the simple fact, that it is much more easy to deprive the blood of its globules than to regenerate them after they have been lost, set an obvious inviolable limit to depletions of blood? and does it not equally reduce to their just value all the exaggerated hopes of hygienic organoplasty? Again, how important is the fact that the numerous forms of the Pyrexiæ—including typhoid fever, although this disease certainly does not exhibit in all its stages a pyrexial character—may be developed without any necessary change in the proportion of the red globules, *while that of the fibrine is never above the normal standard.* This last character alone serves to draw a broad line of demarcation between the genuine Pyrexiæ and the febrile Phlegmasiæ, in which the blood invariably contains a larger proportion than usual of fibrine.

Now, if it is proved that sanguineous depletion forms the most appropriate remedy for the latter class of diseases, will any one venture to say as much for it in the Pyrexiæ, in which there is certainly not the same hematological character? and does not the examination of the blood furnish us with a most valuable and decisive means of ascertaining the existence of genuine inflammation in the course of a Fever?

Then again, the knowledge too of the many anomalous functional disorders, so often attendant upon Anemia, is one of the most important and difficult points of medical practice; when once acquired, it will often serve to guide the physician in some of the most perplexing circumstances of clinical experience.

It is impossible to read, without the most lively interest, all that M. *Andral* has written on the state of the blood in Cancer, Phthisis pulmonalis, and other organic diseases. His experience does not at all confirm the doctrines of the self-called physiological sect, in reference to the development of tubercles. How can we for a moment suppose that Tuberculisation is a mere inflammatory process, when the blood in a patient affected with it is known to exhibit that peculiar alteration, which is essentially characteristic of debility and want of tone? The patient is, in short, in a state of incipient anæmia, and his blood resembles that of a person who has been largely and repeatedly bled. The proportion of the fibrine is found to increase in the course of Phthisis and Cancer, *only* when the tuberculous and cancerous matter has reached the stage of softening or destruction.

In the Neuroses, the proportion of red globules is generally very considerably diminished below the normal standard. This result is strictly in accordance with the therapeutic treatment—viz. the use of tonics—that is most serviceable in such maladies.

The rectifications, which the work of M. *Andral* is so well calculated to establish, indicate a decided movement in favour of the doctrines of medical science, such as it existed before the epoch of the physiological school. The Chloroses, Anemiæ, and Neuroses now resume in the nosological series their place, that was so long usurped by chronic Gastritis, and a host of other subacute irritations; the family of eruptive and other Fevers is separated from that of the genuine Inflammations; and a wide line of distinction is drawn between these and the extensive and varied groupe of organic or structural degenerations.

If, therefore, a decided reaction in medical doctrine has ensued, it may justly be considered as the offspring of the numerous facts, that have been discovered and established by the aid of chemistry and microscopic research.

The more that true science advances, the more distinctly do we perceive that its highest achievements consist rather in an accurate knowledge of the varying conditions of actual phenomena, than in the factitious unity and assumed simplicity of any one doctrine, however ingenious and plausible this may appear to be.—*Gazette Medicale.*

### Researches on the Transmission of Hydatids by Contagion.

Professor *Klencke* of Brunswick, to whom reference was made in the last number of this Journal, (article on Structural and Formative Anatomy,) is the author of the following curious and important observations. They form, in their extended condition, the preliminary chapter to a work which the ingenious author proposes to publish forthwith.

Before narrating the experiments which he has performed with the view of shewing that hydatids may be propagated by transmission from one animal to another, and by their direct ingestion with the food or otherwise into the bodies of living bodies, he describes at some length the various entozoary productions which pass under the generic appellation of Hydatid. The following summary presents the most interesting details of his narrative.

1. *False* or *Spurious Hydatid:* it might be more correctly called *cellula hydropica subindi-viduata.* I have often found it in the brain and the spinal marrow. It essentially consists in the abnormal development of a cellule, which becomes charged with fluid, in consequence of the loss of balance between the powers of exhalation and assimilation. Floating in this fluid, a few globules are often faintly discoverable. Within the primary cellule, other smaller cellules—usually four in number—become developed in course of time; and each of these cellules constitutes a new being or animalcule, which has an independent existence.

2. *Acephalocyst.*—We have no longer to do with simple cellules, which become isolated from the rest of the organism, but with an organised zoological being. They are veritable animals, which have their own peculiar generation. The genuine Acephalocyst is of rare occurrence; most of the cases related by authors belong properly to the preceding species—the false or spurious variety—of Hydatid. The Acephalocyst is a closed vesicle, having a peculiar opaline colour, and varying in size from that of a millet-seed to that of an ordinary pea. The vesicle consists of two membranes, the inner one of which is lined with villosities, which float in the fluid contained within. In the centre of the larger Acephalocysts, there is found a cheesy-like substance which, when examined with a strongly magnifying power, is perceived to consist of numerous minute cellules conglomerated together: these are sometimes seen to exhibit molecular movements under the microscope. The analogy between their structure, and that of the ovaries in different animals, made me suspect that there must be an analogy of function also; and the correctness of this suspicion has been amply confirmed by my subsequent experiments of inoculation with Hydatids. The ovules or microscopic cellules probably escape from the parent cyst by a rupture of the enveloping membranes of the latter. In some instances, the central cheesy matter becomes so indurated that it attains the consistence of plaster or even of ivory : this change may probably be regarded as the result of disease in the entozoary animalculæ.

3. *Echinococcus.*—This parasite is in the form of an antique urn or pear, inclosed in a cyst which is filled with a clear fluid, that is sometimes of a yellowish hue. Its inner surface is found on examination to be sprinkled over with numerous minute corpuscles, which exhibit, under the microscope, a distinctly ampullar form. Some of these float freely about in the fluid, while others are attached to the inner surface of the vesicle—which in the Echinococcus is much thinner than in the Acephalocyst. One extremity of this Entozoon terminates in a disc, furnished with a circle of arms or tentacula, which may be expanded and contracted at will.

4. *Polycephalous Hydatid or Cocnurus.*—This remarkable species consists of irregular vesicles which have several heads, the larger or smaller size of which indicates the different phases of the evolution of the animal. The vesicles may be regarded as so many polypes, each head representing a distinct individual being. As a single vesicle is found to exhibit young, as well as adult, *cænuri*, we are enabled at once to recognise the process of their development. The parent vesicle often presents the appearance of one or more contractions or divisions *par etranglement*—each separate portion probably becoming, in course of time, a distinct animal. The vesicle is in the shape of a leather bag or bottle; the upper or cephalic extremity of which can open and shut at pleasure, while its inferior terminates in a cul-de-sac. * * * * * *

From various considerations I am induced to believe that the polycephalous Hydatids are propagated in two ways; viz. by buds, evolved from their inner surface, and by fission of the parent vesicle; in other words, by gemmiparous and by fissiparous generation. The vesicle must therefore have the power of generating or reproducing, at every point of its surface, buds or new growths, that ere long become capable of an independent existence.

5. *Cysticercus.*—The animalcules of this family are usually of a conical shape, formed in some sort by a cervix and a vesicular body. Within the latter, are to be perceived striæ of minute diaphanous corpuscles, which are in structure analogous to that of the vitreous humour, and in all probability are the ovules of the animal. These corpuscles are sometimes observable on the outer surface also of the parent vesicle.

As Hydatids have been met with in all the tissues of the body, it becomes important to ascertain whether the different species seem to occupy certain tissues in preference to others. As a general remark it may be stated that most of them have been discovered in various organs of the body. As far as we know at present, the only species, whose *habitat* is very much restricted, is the Polycephalous entozoon, which has hitherto not been found in any other part except the Brain. This organ however may be the seat of three other species of Hydatidic animalculæ; the Acephalocyst being regarded as the primary or rudimentary form of the Echinococcus. The false or spurious Hydatid is of most frequent occurrence in the Brain. This simplest form of entozoary production cannot properly be considered as an independent animal existence, which has been introduced into the body from without: most probably it is generated in, and evolved from, the tissue wherein it grows.* I have viewed them, says Dr. K., as cellules detached from the rest of the organism and possessing a sort of semi-independent vitality. This form of Hydatid may be considered as an abnormally amplified organic cell, which continues to follow in its development a different course from that of the regular or normal cells. Once in possession of its individuality, a crop of new vesicles (*blastidies*) resembling the parent one, is often rapidly developed. Although foreign to the adjacent tissues, these hydatids are certainly not so much so as the genuine parasitic entozoa—a circumstance that may perhaps account for the little or no inconvenience that is often experienced during their development. Indeed the only symptoms, that usually accompany their formation in a part, are those attributable to compression. They have been discovered in almost every part of the nervous centres—the substance of both hemispheres, the fornix, the optic thalami, the ventricles, the pons Varoli, between the arachnoid and the brain, &c. It is rare that they are ever agglomerated within one common envelope; usually they are scattered as

---

* This is the view which Mr. *Owen*, in his recent treatise on the Anatomy of Invertebrated Animals, has taken of this question. We may refer our readers to the Review of his work in our last number.

single vesicles, or in small groupes. The symptom, that most commonly attends the presence of false or spurious Hydatids, is a dull *constant* pain in the part: whereas the pain that accompanies the presence of true Hydatids is generally more or less distinctly *periodic.* (?) The only lesion, produced in the surrounding cerebral substance, is a partial atrophy, proportionate to the size of the cell or cells, and consequently to the amount of pressure that has been thereby produced.

With respect to the Etiology of Hydatids, it may be stated that many pathologists have ascribed their formation to the influence of outward injuries, to the suppression of gout or of any discharge to which the system has been long accustomed, &c. It seems not at all improbable that such may be the case with the production of *false* Hydatids; seeing that these causes must obviously tend to promote the disagregation of certain cellules, the one from the other: but we cannot well admit the same explanation with respect to the *true* kind—the developement of which is, in our opinion, always owing to the direct introduction from without of the ovules or germs of the entozoon, and to their subsequent fixation, so to speak, in those parts of the body where they can find a proper *nidus* for their growth. As to the former or spurious Hydatid, it is by no means necessary that it be met with in different parts of the body at the same time; whereas this is very generally the case with respect to the latter species.

The Acephalocysts have been, as we have already said, very generally confounded with the false Hydatids. They are often found in the Brain. It sometimes happens that a cluster of these animals is enveloped in one cyst, with which however they seem to have no immediate connexion. They either float about in the contained fluid, or they sink down in a cluster to the most depending part of the bag. The co-existence of Acephalocysts in the brain, and of Echinococci in the liver, is by no means an unfrequent phenomenon: in such a case, the liver is to be regarded as the primary focus of the morbid production. It certainly seems more than probable that the development of Hydatids within the brain is, in very numerous instances, subsequent to their appearance in other organs of the body.

The polycephalous Hydatid—the presence of which gives rise to the disease known, in the case of herbivorous animals, by the name of the *staggers*—is occasionally met with in the human brain also, producing a somewhat similar train of symptoms. The Cysticercus has likewise been found in this organ, and especially in the choroid Plexus, in the human subject. According to my observations, whenever this Hydatid is present in the Brain, it is simultaneously co-existent in other organs—in which it is usually in a higher and more advanced stage of development.

With respect to the etiology, or the spontaneous development of genuine Hydatids, is there any source or place of abode, we may enquire, without the organism, for the ovules of these entozoary animalcules? They are widely diffused, being found in numerous animals of different sorts; none of them is peculiar to the human species alone. They are transmissible from one creature to another, and may therefore be considered as a living contagious principle, (*contagium animatum.*) It has moreover been observed, that they are not unfrequently voided with the excrements; and no one can now doubt that not only the flesh, but also the blood, the urine, milk, and menstrual fluid, &c. often contain them.

### *The Transmissibility of Hydatids.*

The following experiments serve to throw considerable light on this hitherto unexplored department of pathological investigation: first of the *false Hydatids.*

*Experiment* 1.—Some tepid water, that contained a number of these hydatids collected from a human brain, was injected into the abdominal cavity of several puppies and kittens. After the lapse of three months, the parietal peritoneum was found to adhere to the Epiploon at the seat of, and around, the puncture that had been made with the trocar, and there were numerous false hydatids existing in the neighbourhood of the cicatrix. In one of the kittens, a mass of these productions was found on the peritoneal covering of the urinary bladder, projecting into the cavity of the abdomen.

*Experiment* 2.—A cluster of these vesicles, which were of the size of grains of sand, was injected into the left femoral vein of a young kid. At the same time I mixed a number of them with the milk that was given to a kitten. At the end of three weeks, one of my assistants found in the fæces of the latter animal distinct hydatidiform cellules: I had often looked for them myself, but never could discover any. Six weeks after the date of the experiments, both animals were opened. In the kid there was found in the right (?) groin a hydatidic tumor containing several *cysticerci*:—these might possibly have been introduced in another manner. No trace of any Hydatids was discoverable in the heart or large blood-vessels; but, in the apex of the right lung, I found a largish tubercle, in which, amid the debris of pulmonary cells, there existed one large vesicle, on which there was observable a filiform network all covered over with very minute hydatids. These were given to a sparrow. The excrements of the bird were carefully examined every day, but no trace of the animalcules could be seen. On examining, however, the abdominal viscera, a week afterwards, there was found around the pancreas a mucous mass, which contained several hydatids that were of the same kind as those which the animal had swallowed.

*Experiment* 3.—A number of small hydatidic cells, taken from the brain of a human subject, were inserted into the orbit of an old fowl. At the end of the 13th week after the operation, the orbit was found filled with a cellular mass, which contained a large number of false hydatids. These were all injected into the femoral vein of a young cat. On dissection, three or four weeks subsequently, there was found in the cavity of the heart, and especially at the right auriculo-ventricular orifice, a fibrino-gelatinous mass, which contained an innumerable quantity of false hydatids.

Exceedingly common in the human subject, this kind of Entozoa is much more rare in the bodies of the lower animals.

With respect to the Acephalocysts and Echinococci, I have found the former in the milk of the cow; and, floating beside them in the whey, many of the minute ovules which are met with in the body of these animalcules. On the other hand, it is a matter of almost daily occurrence to discover both these sorts of hydatids in the flesh and blood of different animals.

Dr. *Klencke* has performed several experiments to determine the action of recent gastric juice upon hydatidic animalcules. When pure, it speedily destroyed all traces of life; but, when diluted with water, it seemed to effect only a partial change in their appearance.*

With respect to the propagation and diffusion of the various forms of Hyda-

---

* It would seem, from the result of one experiment, that the Acephalocysts and Echinococci may become, under certain circumstances, transformed and degenerated into a lower and simple form of structure, viz., that of mere vesicles. The following short extract is interesting in more points of view than one. "Two Echinococci, that had been immersed in diluted gastric juice, were introduced under the skin of the abdomen of a dog and allowed to remain there for tidic Animalcules, Dr. K. observes: "As there are continually dying numerous animals whose bodies contain very many Acephalocysts and Echinococci, and as these are eaten by other animals, and the refuse discharged by the excrements, we may readily understand that there must always be a vast number of these beings in the external world—whether in the state of ovules, or in that of individuals more or less completely developed. These individuals, when they are admitted into the human intestinal canal, may become fixed and attached by means of their sharp extremities, and subsequently they may work out a passage for themselves from the bowels into the substance of the different tissues. On this principle, we may perhaps explain how it comes to pass that, in certain localities, the Echinococci are, so to speak, endemic—in consequence of the property, which the waters of these places may have in promoting the development of their ovules. As these ovules are excessively minute, they may, when once received into the torrent of the circulation, be readily transported to any part of the body. The ceaseless movement of the blood prevents them from being stationary or fixed, so that the body of a living animal may always have a number existing in the circulation; unless, from some cause or other, they become adherent to the parietes of the vessels, or are detained in the substance of the tissues.

If it be admitted that the ovules of Hydatids may circulate along with the blood, we shall cease to wonder so much at the not unfrequent appearance of these productions after wounds and other external injuries.

The general conclusions which Dr. K. has drawn from his numerous experiments, are these:—

1. That, in all the species of Hydatidic Animals, the mode of generation is two-fold, fissiparous and oviparous.

2. That there are false or spurious Hydatids, which are propagated by *blastidia.*

3. That all the different sorts of Hydatids are communicable from one organism to another; and, as they are found to exist in fluid food, and in the flesh of different animals, they may be readily transmitted by infection.

4. That the Acephalocysts are not distinct from the Echinococci; the former are only the ova of the latter, with or without the parent envelope.

5. That the current of the circulation serves to diffuse the Hydatidic Animalcules, whatever be the mode in which they have been introduced into the system.

6. That there exist agents in the living organism, as well as numerous substances in the Materia Medica, which are capable of acting as poisons to these parasitic productions.—*Gazette Medicale.*

---

three weeks. On then examining the part, there was found a cellular cavity, which contained a yellow serosity, in which floated the two Echinococci, 'notably modified.' They had become transformed into vesicles, the outward surface of which was covered with a multitude of buds and isolated cellules supported on short pedicles. Under the microscope, these cellules, when squeezed, were observed to give out a multitude of minute cellules, like to those which are found in the bodies of Acephalocysts, and which may be regarded as ovules. When the Hydatids were laid open, their inner surface was observed to be sprinkled over with a still greater number of buds and pediculated vesicles, while others floated in the contained fluid."

### Exopthalmia from an Hydatid within the Orbit.

The case occurred in a boy, eleven years of age. The left eyeball was fairly protruded from the socket, so that the organ was only partially covered by the distended lids, whose edges, being turned inwards, kept up a constant irritation. In consequence of this exposure, the cornea had become somewhat opaque, and the impairment of vision still more considerable. The pains, which the patient experienced, seemed to be produced almost entirely by the distention and compression of the affected parts. The eyeball had become very gradually displaced; the parents of the boy having observed the deformity more or less distinctly for more than two years. The organ itself was not enlarged; and indeed it was quite obvious that the protrusion was owing to a tumor, situated at the outer side of the orbit, covered by the swollen and injected conjunctival membrane. It was firm, but elastic, to the touch; and, when pressed on, it communicated to the fingers the sensation of a fluid being contained within. I felt satisfied, therefore, that the case was one, not of a solid, but of an encysted, swelling. Moreover it could not well be of a meliceric or atheromatous nature; for it was developed at the bottom of the orbit, at a distance from any sebaceous crypts, and it had no relations of continuity except with the skin and the conjunctiva. The diagnostic question came therefore to be, was the cyst a purulent, or was it a serous, one? There had been no antecedent symptoms at any time to warrant the belief that suppuration had ever taken place in the part affected. I therefore concluded that we had to do with a serous cyst or hydatidic formation, that had become developed within the orbit; and this conclusion was a good deal confirmed in my mind by remembering a case of this, that occurred in the Clinique of the Hôtel Dieu in 1828. In that case, the tumor did not project much at the side of the orbit, so that it was at first suspected that the eyeball itself was the seat of the enlargement; and extirpation of the organ was therefore recommended and performed. On the first stroke of the bistoury, an aqueous fluid streamed out from the bottom of the orbit; the operation was continued; and, on removing the eye, an hydatid fell out on the cheek of the patient: the eyeball itself was not at all diseased. In the present instance, I determined to proceed with a good deal of caution. On cutting through the conjunctiva over the tumor, a quantity of limpid serum flowed out, and the protrusion immediately subsided very considerably. When the edges of the wound were separated, a white puckered membraniform substance was observed at the bottom of the pouch from which the fluid had escaped. On removing this with a forceps, it proved to be a solitary hydatid, which, when distended, must have been as large as a full-sized walnut. The eyeball at once retired back within the orbit, still retaining however the oblique direction outwards, which it had before. This partial squint remained, when the wound had quite healed.—*La Clinique de Marseille.*

---

### Microscopic Entozoa in the Blood, Stomach, &c.

Modern microscopists have demonstrated, beyond all doubt, the existence of living animalcules in the blood of various animals. All those, heretofore discovered, were found to belong to the genus *Filaria*. It remained to be shewn whether there was not some variety in the sanguineous, as there is in the intestinal, Entozoa; and also whether the presence of such parasitic creatures should be ever regarded as a normal, or only as a pathological, phenomenon. With this twofold object in view, Mr. *Gruby* has recently devoted himself to an extensive series of researches; one curious result of which has been, to discover in the blood of frogs a new species of animalcules, as remarkable for their shape as for their movements. They are found in the blood of the animal during the seasons of Spring and Summer. The body of this blood-Entozoon, is convoluted like an auger; hence M. *Gruby* proposes to call it *trypanosome*—*τρυπανον*, terebra, and *σωμα*, corpus. Not improbably he is mistaken on this point; as it seems more likely that the twisted appearance is owing to the writhings of the animalcule than to its natural configuration. The *Tripanosomes* are certainly not nearly so common in the blood of the frog as the Filariæ; our author did not find them in more than in two or three out of a hundred animals which he examined. They seem to be more frequent in the blood of the female than of the male animal.

These observations, taken in connexion with those of *Valentin* and *Gluge*, establish beyond all controversy the existence of different species of Entozoa in the blood of cold-blooded animals. M. *Gruby* is inclined to believe that they are not to be viewed as the result of any diseased process in the body of the frog, but that they are normally present in the blood, under certain unknown conditions of the system. But it is not in the blood only that they are found; they have been observed in the visceral cavities of the frog, and also in the bodies of other animals higher in the scale of zoological organisation.

*Intestinal Entozoa during Digestion.*

In 1685, *Leuwenhoeck* announced that he had discovered three species of microscopic animalcules in the excrements of frogs, birds, and even of man; but the truth of this statement was till of late years called in question by *Ehrenberg*. Although the perfect accuracy of the father of microscopic physiology has been now amply confirmed, no one seems ever to have suspected the existence of living animalcules in the stomach and intestinal canal during the process of digestion in the higher animals, until the recent researches of MM. *Gruby* and *Delafond*. According to the observations of these gentlemen, it would seem that there are no fewer than four different species of living microscopic animalcules discoverable in two of the stomachs—viz. the paunch and the bonnet—of ruminant animals, while the process of digestion is going on. The transparency of the enveloping membrane of these gastric Entozoa permits the observer to see the molecules of food which they draw in, and the ingestion of which renders their bodies more or less opaque at first. The number of these animals is so great, that, in five centigrammes (less than one grain) of the contents of the first and second stomachs of a sheep, there have been found at least 15 or 20 animalcules of different species, and varying too from each other in size and configuration. MM. *Gruby* and *Delafond* estimate that they constitute nearly one-fifth of the entire weight of the alimentary matter, in which they are found. In the third and fourth stomachs—viz. the manyplies and the red—the animalcules are found to be dead, and can be recognised only by their *carapace*—the enveloping or tegumentary covering of their bodies—having become quite empty and transparent. In the small and large intestines, no traces, save only some of the debris of the *carapaces*, are discoverable of the gastric Entozoa.

MM. *Gruby* and *Delafond* have discovered no fewer than seven different species of animalcules in the colon of the horse If it be asked, what possibly can be the use of this ever-renewed development of animalcular life in the alimentary canal of herbivorous animals more especially, these gentlemen reply, that they serve to *animalise* the vegetable food, and fit it for becoming assimilated with the blood and solid tissues of the body. We have seen that they calculate that not less than a fifth part of the entire chymous mass becomes transformed, so to speak, into a congeries of the most-simply organised animalcules—which, being digested in their turn, are supposed to furnish a supply of animalised matter for the nutrition of the animal in whose body they exist. The food of the dog and swine being much more highly animalised, the development of digestive Entozoa is not necessary in the same degree: hence they are much fewer in number and less varied in form in these, than in herbivorous, animals.

These statements are certainly highly curious and interesting; but the question comes to be, are they altogether correct and trustworthy? There are so many sources of deception in the microscopic examination of living matter, that we must be very cautious in receiving as *proved,* the startling announcements that are every now and then brought forward.—*Gazette Medicale.*

---

### CHEMICAL PATHOLOGY.

The subsequent remarks may be considered rather fanciful by many readers; but they at least deserve notice, as indicating, among other events of the present day, the marked tendency that now exists to a recurrence to a Chemico-Medical system of nosological interpretation.

" It may be readily imagined that many diseases must have their cause and origin in certain anomalies or derangements in the process of the metamorphosis of *proteine* into the different elementary constituents of the body, as the albumen, the fibrine, caseine, and globuline. Sometimes it is the non-azotised, at other times it is the azotised substances, whose properties undergo such an abnormal alteration. We may take, for example, a class of diseases, which in their essential nature differ from each other, only in as far as their influence and operation are more or less widely extended over the one or the other of these elementary bases of the animal economy—we allude to that section of the nosological catalogue that comprehends such maladies as tubercles, scrofula, rickets, arthritis, and osteo-malacia. The first two of these diseases are characterised by an abnormal metamorphosis of the azotised materials of the body. In tuberculous disease, there is an imperfect metamorphosis of the Albumen, which does not advance, so to speak, beyond the formation of Fibrine. From what we have stated on a former occasion, there is every reason to believe that it is in consequence of the union of the oxygen of the respired air with the Proteine that the constituent principles of our bodies are primarily formed. In *tuberculosis* we observe that the oxygen, from some causes hitherto undiscovered, does not so perfectly unite with the Proteine as to metamorphose it completely into an animal tissue. A somewhat similar thing occurs in *inflammation.* The oxygen, which passes into the blood, unites with only one atom of the sulphur of the albumen, and forms fibrine; and this element thus accumulates in the blood, in consequence of its not being sufficiently oxydised either to form organic structure, or to be separated by the process of excretion. Hence the fibrinous condition of the circulating fluid in the phlegmasiæ, and hence too the formation of false mambranes, and other similar products. In *tuberculosis,* the blood is equally charged with non-oxydised combinations of Proteine; and, as the oxygen does not render them excretable by the usual channels, the system gets rid of them by causing their deposition in those parts of the body whose tissue is most lax and yielding. *Scrofulosis* differs from tuberculosis only in being generally a disease of childhood—the age when the plasticity of the organism is the greatest. Hence, in the former malady, there is a greater amount of imperfectly oxydised Proteine continually in requisition, so to speak; and hence, too, there is less frequently the formation of morbid deposits of this substance in the parenchymatous tissue of organs than the separation of non-azotised materials by abnormal and extraordinary outlets." * * * * *

" Pathological observations shew that the fat plays an important part in the production and continuance of many diseases. The opinion of *Liebig,* that the white and oleaginous state of the blood, which is occasionally found to exist in drunkards, is owing to an incomplete oxydation of the fat—in consequence of the combustion of the alcohol,—appears to be rather far-fetched, Is it not more probable that it arises from the morbid state of the liver, that is so very commonly present in persons of intemperate habits? It is unquestionable that the secretion of the bile has an intimate relation with the condition of the fat of the body, so much so, that whenever the former function is inactive from induration or any other morbid lesion of the liver, the absorption of the fat in the body is invariably observed to go on very slowly. We have not unfrequently had an opportunity of observing the truth of this remark in certain cases of Phthisis; the emaciation under such circumstances being very gradual indeed, if the liver has become at all indurated. We know too that very corpulent people are usually subject to hepatic derangements; and it is a common remark that patients, recovering from Typhus fever, are apt to become unusually lusty."—*Archives de Medecine.*

---

### On the Use of Ptisans in France.

The following very sensible observations on the use of ptisans, and on some other points in French medical practice, are from the pen of Dr. Higgins, an English physician who has resided for upwards of 15 years in France, and who, we may therefore suppose, is tolerably well qualified to give an opinion on the subject that he discusses. They are contained in a letter recently addressed by him to the Gazette Medicale, the editor of which, while claiming the indulgence of his readers for the language of a stranger, expresses his own and their obligations for "les excellentes remarques sur la pratique des deux pays"—France and England.

Without any circumlocution, the doctor at once announces the object of his writing.

"I propose," says he, "in this letter, to attack the use of ptisans, which I regard, with but few exceptions, as utterly useless, and indeed as often positively injurious."

During the course of a disease, whether this be of an acute or a chronic nature, the ingestion of a ptisan is regarded by the French as a necessary part of therapeutic regime, and is very generally viewed rather as a direct and efficacious medicinal agent, than as a mere diluent beverage. Not a few practitioners seem to approve of the custom, without having any very distinct or well-grounded faith in the curative powers of the remedy.

The number of ptisans used in France is very considerable; and consequently it is often not a little embarrassing to the neophyte to become thoroughly acquainted with all the minutiæ of a sick chamber's regime in France.

Cross the Channel, and what a contrast!—a penury of ptisans that is really distressing. As the great *Careme* said of the English, that they had but one sauce—melted butter—so it may be truly said that they have but one ptisan—barley-water! In England the indifference of physicians, on the subject of the drink to be used by their patients, is quite as great an error as the over-concern of their French brethren on this point of practice. The English prefer the use of potent and promptly-acting remedies from the shop of the chemist; while the French choose rather to trust to time and to the simples of the herborist. Curious subject of speculation to the physiologist!—the Frenchman so mobile and vivacious in health and yet so patient in sickness; and the Englishman so phlegmatic in the one, and withal so impatient of delay in the other.

In France they make use of four sorts of ptisans—diaphoretic, diuretic, diluent, and aperitive. The *first* are used whenever it is wished to promote the cutaneous perspiration, or the eruption of an exanthematous eruption. These results are much encouraged by keeping a warm dry air around the patients' body at the same time. The *second* are indicated when the object is to increase the flow of urine; and the *third*, when we have reason to believe that the fluids and secretions of the body are more than usually acrid:—hence the utility of these latter in almost all febrile and inflammatory affections.

In my opinion, the employment of the diluent and aperitive ptisans is liable to very great abuse in French practice. How very commonly are indigestion, and, its ordinary consequence, constipation, induced by their excessive employment; and what a host of ills is comprised under these two simple words! Put Dyspepsia on one side, and every other malady of the nosological catalogue on the other, and the former will predominate in point of frequency.

The biliary secretion, like that of the other chylopoietic viscera, is invariably altered and modified, whenever the digestive process becomes deranged. Then follow either constipation or a tendency to diarrhœa, accompanied with a furred state of the tongue, an offensiveness of the breath, and a multitude of other vexatious symptoms.

Now if this state of things should result from an inflammation of any of the abdominal viscera, the treatment, every one knows, pursued by the English and French physician is very nearly the same: recourse must be had to bleeding and other depletory and antiphlogistic measures, before proceeding farther; but, arrived at the delicate point of the arrest of the inflammatory action, the practitioners of the two countries will be found to diverge most widely in their practice. In France, the patient is kept for some days on diluent ptisans, before aperient medicines are administered; while in England no time is lost, but recourse is had at once to the use of purgatives, followed perhaps by that of diaphoretics.

At a period, when the Medical Constitution is inflammatory (as was the case when *Broussais*' doctrines were in the ascendant) the French practice is unquestionably to be preferred; but, in the present hygienic conditions, most unprejudiced men will prefer, I should think, that of the English physicians. Sufficient attention is not paid in the present day to the various conditions which are so frequently occurring in the *medical constitutions* of the seasons; and yet nothing is more certain than the fact of these alterations and vicissitudes.

*Tempora mutantur, et nos mutamur ab illis.*

At the beginning of the present century, the publication of Dr. *Hamilton's* (of Edinburgh) work on Purgatives gave rise to a very marked and important change in the medical treatment of many diseases. So satisfactory were the results then obtained by most English physicians from the judicious administration of this class of medicines that, when, a few years after, the genius of *Broussais* perceived the arrival of the inflammatory Constitution, they refused all assent to his doctrines, and would not even give a fair trial to the treatment which he recommended.

In France, on the other hand, the new system of pathology was received with the warmest enthusiasm; the younger physicians adopting it with an almost blind credulity, and their elders finding in it a wherewithal to modify, in some respects, the practice which they had long been accustomed to pursue.

But we have now arrived at the stumbling-block of our enquiries; for it can-be questioned by any one that is conversant with general practice that, for several years past, the Inflammatory Constitution of the seasons has ceased to exist, and has been replaced by that which preceded it; viz. by one of a catarrhal and humoral character.

The French, before the time of *Broussais*, did not use purgatives to nearly the same extent as the English; and the opinions of this celebrated man contributed not a little to restrict their use still more, and to introduce the very general substitution of diluent and gently aperitive ptisans. This system—although certainly carried to the same excess in France, as that recommended by Dr. Hamilton has been in England,—produced so very favourable results for a certain time, that the Continental physicians cannot, even yet, make up their minds to discontinue it, although the medical Constitution, which gave rise to it, has unquestionably ceased to exist—so that we may with equal justice blame the English physician of 15 or 20 years ago, and the French one of the present day, for very similar faults.

Gastritis and Gastro-enteritis are infinitely less common *now* than they were *then;* while simple gastric derangements, accompanied with a vitiated state of the secretions, are much more frequent. In short, Dyspepsia, as we have already said, is *the* disease of the present day; this is the genuine *fons et origo* of ill-health in most instances, when the patient fancies his malady is seated elsewhere; and it is for *it* that a physician is consulted in three, out of every four, cases that occur in his practice. Dyspepsia complicates almost every morbid affection, from a simple boil to grave typhus fever, from a mere scratch to the most important surgical operation.

In such a case, if the Broussaian practice be adopted, the patient will be kept on the sick list for two, three, or more weeks, whereas one or two doses of a brisk purgative might have relieved him in as many days. So much for adhering to a mode of treatment, while the medical Constitution (which at first demanded its use) has passed away. Among the leading physicians in the French metropolis, there has been, for some years past, a manifest secession from the Broussaian creed; most of them begin to recognise the advent of another reign, without however having entirely rid themselves of all their former prejudices.

Whenever, indeed, we have reason to believe that a genuine Phlegmasia of the stomach or intestines does exist, we should certainly trust to local bleeding and the use of cataplasms and emolient ptisans, avoiding the use of purgatives, until the inflammatory symptoms have subsided. But then comes the question, how are we to determine this period precisely?—here is a point on which the English and French physicians are greatly at issue. The one is for temporising and delay; the other is for prompt action. The one is afraid of rekindling the the smothered excitement and of being obliged again to have recourse to depletory measures; the other, well aware of the immense relief generally experienced by a free evacuation of the bowels, is impatient to administer his favourite remedies. Twenty years ago, the English physician unquestionably carried the purgative treatment to an injudicious length; but, since that time, the doctrines and precepts of the Broussaian school have taught him to be more discriminating in his practice.

I must confess that I have never been able to understand the reason of the great aversion of French practitioners from the use of brisk purgatives. Often after the application of leeches, cataplasms, and emollient ptisans for days, or even weeks at a time, and when these means have quite failed in giving decided relief to the patient—instead of exhibiting a good active purging dose—recourse is had to a caustic issue in the arm or somewhere else! and this disgusting remedy is indeed so generally adopted in Continental practice, that we may almost always snuff the unpleasant odour of a purulent discharge, when travelling in public vehicles in France!

The mere circumstance of there being some amount of tenderness of the abdomen, on pressure, is not in itself a sufficient counter-indication of the use of purgative medicines. Take for example a case of typhoid Fever, in which this symptom almost always exists to a ceetain degree, along with a greater or less degree of disturbance in the circulation. And yet, many of the best French physicians are in the habit of administering purgatives in this malady with the most satisfactory results. Of this I am certain that, in a multitude of cases, more good will be done in the course of a week by the judicious use of purgatives, than can be effected in a month by a succession of inert ptisans, and troublesome blisters and caustic issues. The *derivation,* caused by the action of the former along the whole length of the intestinal tube is much more potent, and moreover much more in accordance with the indications of Nature, than what can be produced in any other way: for where can we find a revulsion equal to that which sets a working the innumerable glands of the bowels, as well as the liver and other chylopoietic viscera?

No one can deny that the use of Mercury, as a purgative, has been very indiscriminately resorted to by English practitioners; but things are somewhat altered now; and there is no good ground for the prejudice that exists in the minds of almost every French physician, that calomel is administered by us on all occasions, and under all circumstances, whatever be the nature of the malady that is present, or the character of the patient's constitution. The first question that a French doctor usually puts to a patient, who has been under treatment by an English one, is, "Ah, ah! you have taken calomel; is it not so?" And, ten chances to one, he forthwith attributes all the ills of his client to his having been poisoned with enormous successive doses—20 or 30 grains at a time—of mercury. This error is mainly attributable to the exceeding ignorance of most French practitioners as to the state of medical literature in foreign countries. Their journals make few extracts from the English ones; and those, that are made, are seldom of that kind to give a good idea of the practice on the other side of the Channel.

Few French physicians are willing to admit that the preparations of mercury have any specific action on the biliary secretion. On this point they are egregiously in error. As well might they deny, in my opinion, the specific action of bark in ague, sulphur in scabies, or ergot of rye in producing uterine contractions. Mercury, and especially calomel, is one of the most valuable articles of the Materia Medica; and, when administered with judgment and discretion, will often effect more benefit, in the course of two or three days, in dyspepsia and other derangements of the stomach and bowels, than all the ptisans in the world, though these are persevered in for several weeks at a time.

One word as to the amount of doses applicable to the two people, to which we have been alluding in the preceding remarks. The French physician is often astonished, if not affrighted, at the large doses of certain potent medicines which are so commonly administered without reserve in English practice: to a certain extent, there is certainly some ground for this alarm. The French are on the whole not so robust as the English; their diet is not so nourishing; and the air of their climate (Normandy, Picardy and Brittany excepted) is drier and more elastic. For these reasons, they cannot bear such strong and drastic doses. Perhaps about one-third more of any medicine may, as a general rule, be administered to the latter, than to the former. But surely no one can believe that there is such a difference between the climates, customs, and constitutions of the two nations, as to require an essentially different mode of treatment in the same, or in similar, maladies. And yet this idea has been long entertained by not a few medical men on the Continent. Fortunately, however, this prejudice—for we can regard it as nothing else—is gradually disappearing; and the practice of all countries begins to be more and more distinctly based on the same principles.

---

### M. REMAK ON MENSTRUATION.

"*Aristotle* asserts that Viviparous quadrupeds are, like the females of the human race, subject to a periodic discharge of blood from the organs of generation; although the quantity indeed is very much less in the former than in the latter. He adds that, the cessation of this discharge in mares and cows is a sign of conception having taken place. He calculates that the amount, lost by a cow during *heat*, must be about the measure of a 'semihemia.' *Pliny*, on the contrary, maintains that there is nothing at all analogous to the menstrual function in the lower animals. *Haller* was of a similar opinion with the Roman naturalist; and so was *Blumenbach,* who regarded the alleged catamenial secretion of monkeys to be a discharge of very irregular and only occasional occurrence. On the other hand, *Buffon* and *Frederick Cuvier* most distinctly maintain that certain mammiferous animals are subject to a periodic discharge of blood from their generative organs. According to the latter naturalist, the males of quadrumanous animals —when in a state of health and kept in captivity—experience an almost continual *besoin* of propagation, while their females are subject to this impulse only at determinate periods or epochs. When they (the females) are in *heat,* then there is observable a discharge from the vulva of a sanguineous fluid, which has sometimes all the characters of a veritable menstruation. The animal will not receive the approaches of the male, unless she be in *heat* at the time; the periods of which usually return every 20th or 30th day.

No *heat* or venereal excitation is experienced during the period of gestation. F. Cuvier has found this to hold true in many species of the Simiæ. He has also observed a menstrual sanguineous discharge—although its periodic recurrence was certainly more irregular than in the Quadrumana—in the mare, swine, and buffalo. *Meckel* tells us that he once noticed in a female monkey a copious periodic discharge of blood, which returned very regularly every month, and which lasted for several days each time. *Cableis* has observed in cows a mensual flux, coincident with the recurrence of *heat* in the animal; and *Ehrenberg* makes a similar remark in reference to many of the Quadrumana.

In 1838, *Numan* published some very interesting observations on the periodic discharges of blood from the generative organs in some of our domestic animals, and more especially in cows. He found that, very often at least, if not always, this flux takes place in the cow at the season of *heat,* and that both these phenomena or physiological conditions usually recur every 19 or 20 days. The discharge ceases not only during gestation, but also during the greater part of the period of suckling. Generally it does not make its appearance until the 2nd or 3rd day of *heat,* when the sexual feelings have become strongest. The discharge does not take place in a continuous, but in an intermittent, manner; every now and then, and at variable intervals, there is an issue of a sanguineous character from the vagina. The quantity lost seldom exceeds one or two ounces at most; the blood has a bright vermilion colour; sometimes it is pure and liquid, and at other times it is mixed with a good deal of mucus. The discharge usually lasts for two or three days at a time. *Numan* has not observed a similar phenomenon in mares: in them, as well as in sheep and swine, there is a discharge of mucus only from the vagina during the periods of *heat.* The mucus is indeed occasionally observed to be streaked with blood; but this admixture may be owing to a slight abrasion of the parts produced by over-frequent copulation. To determine with accuracy the part whence the sanguineous discharge from animals in a state of *heat* proceeds, *Numan* made a careful examination of the generative organs of a cow that was killed during this period. The mucous coat of the vagina he found to be highly reddened, but there was no trace of oozing of blood from any part of its surface; although there were indeed present a few oblong soft coagula, that appeared to have come down from the uterus. On opening this organ however, its entire inner surface, even as far as the cornua, was found to be covered with red blood, while its cavity was partially filled with coagula. The blood seemed to have proceeded exclusively from the uterine caruncles, of which there were visible at least sixty or more, of various sizes—from that of a pea to that of a kidney-bean.

From these and other observations, we may fairly conclude that, in the females of several mammiferous animals, a periodic discharge of blood really does take place from the generative organs. Whether we should consider this discharge to be analogous to the menstrual flux in women is, however, very doubtful; for in the one case it is obviously and invariably connected with an existing venereal orgasm of these parts, whereas, in the other, it does not seem to be so. The rutting of animals is manifestly the periodic renewal of sexual activity, internal as well as external; and this is evidenced by the desire for copulation on the one hand, and by the aptitude for generation on the other; whereas, in women, on the contrary, the act of menstruation is merely a periodic increase or exaltation of the continued activity of the internal organs of generation, the aptitude for conception being present at all times, and being only more decided immediately before and after each catamenial period.

Although we cannot readily admit that the monthly sanguineous discharge is an accidental phenomenon, which is not necessarily associated with the function of menstruation, we are nevertheless led, by a careful examination of numerous physiological facts, to the conclusion—one too which has generally been adopted by the best writers—that the mere discharge from the vagina does not constitute in itself the essence, so to speak, of the function of Menstruation; but that this is only an outward evidence of a change, that has already taken place in some internal part or another of the generative organs. The non-appearance of the catamenia in a girl, about the period of puberty, is not to be regarded in itself as an actual disease, but only as a sign of atony and feebleness of the system to develop the ovarian vesicles. Hence the imprudence of those practitioners who, when the state of a patient's health at this period becomes deranged, immediately resort to the use of remedies with the view of inducing, or rather forcing, the catamenial discharge, as if the absence of this evacuation was the only and real cause of all the mischief.

Although a patient in this condition is indeed almost always much better when the discharge is once fairly established, all that we can fairly infer from this circumstance is, that its occurrence is only a sign or evidence of the improvement of the general health, quite independently of the relief which a local drain of blood will often produce in certain diseased states of the body. The establishment of the menstrual flux acts as an innocuous mode of relief for other anomalies of the health, which are not unfrequently referrible to the action of the generative organs; but it is far otherwise with the suppression of the discharge, after this has been once long established. The cessation of this compensating evacuation is apt to induce troublesome consequences, which often compromise, in a very serious manner, the health during the remainder of life."—*Annales de la Chir. Française et Etrangère.*

---

### M. RACIBORSKI ON THE CORPORA LUTEA.

"The result of all my researches has been to leave no doubt on my mind that the *Corpora lutea* are in fact nothing else but mere modifications of the ovarian Follicles, from the highest or most advanced degree of their development to the formation of those yellow-coloured tubercles which we find in many animals, and the yellowish spots which are observed in the human subject also, and which are in truth the traces or vestiges of ancient follicles.

"All these varieties or degrees of structural development have been very confusedly grouped together and characterised as the *Corpora lutea* of the ovaries; but most frequently this appellation has been employed to designate that degree or stage, which follows immediately upon the rupture of the follicles. Now the fleshy masses, which we meet with, at this stage of development, in the lower animals, are produced by the folding or plaiting of the internal membrane of the vesicles which have become diminished under the influence of the retraction of the external tunic, and of the reciprocal adhesion of the circumvolutions. As to the spongy masses of a reddish hue, which we find in the ovary of the human female at this period, we may with confidence assert that they are formed by the fibrinous coagula of blood, and are destined to play the same part in the economy of the human female as the fleshy-looking masses do in that of the lower animals.

"Some writers have described two sorts of Corpora lutea, the true and false;—the first only being regarded as the consequence of actual conception. This opinion is, however, very far from being yet established as correct. Numerous observations on animals have now satisfied me that the appearances, found in the ovaria from rupture of the Graafian vesicles, are entirely similar on all occasions, whether this rupture may have been preceded by sexual connection or not. The experienced pathologist will not confound the alterations which are produced by rupture of the vesicles—in other words, the proper Corpora lutea—with other ovarian appearances which may have some resemblance to them.

"The number of Corpora lutea is almost always equal to that of the fœtuses contained in the uterus. Thus there is usually but one in uniparous, and two or more in multiparous conceptions. Still, it is not rare to find in the latter case a greater number of the yellow than of the minute bodies. This may be owing to the circumstance that several of the ovules—probably those, whose 'ponte' has been somewhat retarded—have not been fecundated. Having never, on any occasion, found fewer Graafian vesicles than fœtuses, I think myself fully warranted in recognising in this fact the expression of a law, that holds good throughout Nature, and in believing that the same must be the case in multiparous conceptions in the human female, and that there are never two ovules present in one and the same Graafian follicle.

"We need scarcely say that, in all examinations of this sort, it is most important,—when we endeavour to determine the relations that exist between the number of the Corpora lutea and that of the fœtuses,—to recollect that, in the presence of every gestation, there are always the Corpora lutea of preceding 'pontes,' and consequently that it is most necessary to distinguish those which belong to the last pregnancy from all the others."—*L'Experience.*

(Would that many of the descriptions and narratives of continental writers were often more easily intelligible! As far as we understand M. *Raciborski's* Essay, we presume that, in his opinion, the Corpora lutea are certainly not produced by conception, and consequently cannot be any sign or mark of impregnation; but that they are the mere result of the rupture of the ovarian vesicles—an act or process that takes place at every period of menstruation.)

---

### On Tympanitis and Dropsy of the Uterus.

Those distinguished obstetrical authorities, *Stoltz* and *Naegele,* having, at the Medical Congress held in 1842 at Strasbourg, expressed their unbelief in the existence of these diseases, Dr. *Tessier* of Lyons has collected together the details of several cases which, he thinks, clearly demonstrate the error of their opinion.

*Case* 1.—A middle-aged woman, of a strong but highly nervous constitution, was admitted into the Hôtel Dieu, for chronic metritis complicated with Hysteria. The body and cervix of the uterus were found to be considerably engorged on manual examination; there was present an old-existing leucorrhœal discharge; the catamenia were irregular in their recurrence; and the patient complained of pain in the hypogastric and renal regions. About the period of her reception into the hospital, the abdomen began to become much tumefied, and the quantity of leucorrheal discharge to be very decidedly diminished. As the menses were also absent, the woman believed herself to be pregnant. On vaginal examination, the uterus was found to be voluminous, but light; while percussion of the hypogastrium elicited a clear sonorous sound. The distention of the abdomen gradually increased, and the woman began to fancy that she felt the movements of the child within her; but, as neither the placentary bruit nor the sound of the fœtal heart could be detected on auscultation, (the menses had now been absent for more than six months,) I was of opinion that she was not pregnant at all. One afternoon she was suddenly seized with labour-like pains, and these were quickly followed by a copious discharge of an offensive *flatus* from the vagina. As this escaped, the size of the abdomen gradually subsided, until it was nearly flat. It should be remarked that there was no clot, mole, or any other substance expelled, the putrefaction of which could at all account for the gaseous secretion. This woman was subsequently liable to returns of uterine Tympanitis, which always relieved itself in the way now mentioned. This case occurred in Dr. *Tessier's* own practice, and he had therefore abundant opportunities of ascertaining the perfect truth of all the particulars. He proceeds to cite other cases reported in various works. *Frank* (in his Medicina Practica) mentions the cases of two women, who, after being long subject to leucorrhea, were every now and then seized with hypogastric pain, tenesmus of the uterus, sickness, and tendency to syncope: these symptoms generally vanished upon the explosion of a quantity of gas from the vagina. He alludes also to the history of two ladies, who were believed to be pregnant by more than one eminent accoucheur, but in whom the abdominal distention was owing entirely to uterine Emphysema.

In the Revue Medicale for 1830, an account is given of a lady, who supposed that she was five months advanced in pregnancy, in consequence of the cessation of the catamenia, and the gradual enlargement of the abdomen. Her hopes, however, proved vain; and all symptoms vanished upon the escape of a quantity of flatulence from the vagina. M. *Colombat* relates a similar instance in his Treatise on the Diseases of Women. M. *Lisfranc* also, in the 3rd volume of his Clinique Chirurgicale, mentions the case of uterine Tympanitis; but, in this instance, there was also a mole present—although a small one—at the same time; so that this cannot be regarded as a genuine example of the disease.

So much for Tympanitis of the uterus; let us now briefly notice some cases of Hydrometra or Dropsy of this organ. *Fernel* tells us of a woman, who, at each menstrual period, discharged by the vagina an enormous quantity of water, upon the escape of which the abdomen subsided, and the menses then flowed as in health. The serous collection formed regularly every month. *Mauriceau,* in reference to this malady, expresses himself thus:—"There are women who, believing themselves to be pregnant, have only dropsy of the uterus. This was the case with a patient whom I saw some years ago. She never was pregnant; but she continued to entertain hopes of a family till she was 55 years of age, because there remained, up to that period, occasional traces of menstruation. On one occasion, she was so fully convinced of her approaching accouchement, that she prepared the baby's clothes, and actually sent for her nurse to be with her. Two or three days afterwards, to her great disappointment, all her hopes vanished in a discharge of wind and water from the uterus."

In the 2d volume of the Journal de Medecine, there is the report of an interesting case by M. *Blegny,* of a Countess who was affected, for several years, with a distention of the abdomen, and some other symptoms which at first had given rise to the suspicion of pregnancy. During a fit of violent coughing, she discharged a prodigious quantity of water: from that day she never ailed a whit. M. *Lisfranc* also may be adduced as admitting the occasional existence of Hydrometra, and he cites some interesting examples of it. We may briefly notice one of them.

A woman, 35 years of age, who had been long subject to catamenial irregularities, became affected with dropsy of the uterus. A variety of remedial means —such as drastic purgatives, emetics, sternutatories, &c.—were tried, but without avail. An elastic-gum bougie was therefore introduced into the cavity of the uterus, and immediately a quantity of watery fluid escaped. The uterine distention at once subsided, and the patient quickly regained her health.

*Vesalius* tells us that he once found in the uterus, after death, a collection of serosity so considerable, that it could not be less than 90 kilogrammes, (plus quam centum et octoginta libras aquæ serosæ.) *Nicolai* relates a case of a sexagenarian widow, in whom the uterus was so much distended as to reach nearly to the zyphoid cartilage; the os tincæ was quite closed; on opening it, six measures at least of a fluid, like the lees of wine, flowed out.—*Gazette Medicale.*

---

### On the Good Effects of Opiates in Metrorrhagia.

Whether M. *Simon* be correct, or not, in his idea that many cases of Metrorrhagia are dependent upon an undue nervous excitement of the uterus and its appendages, we entirely coincide with him in his laudation of opiates to arrest hæmorrhage from this organ. In a few cases, indeed, it may be necessary to have recourse to blood-letting and other antiphlogistic remedies, as in very plethoric and inflammatory subjects; but such instances form the exception and not the general rule. The French accoucheurs have, it may be observed, very generally manifested a marked disinclination to administer opiates in uterine hæmorrhage—whether this occurs at the menstrual periods, or whether it happens before or after child-birth and abortion. The following case may be mentioned as presenting a sample of the injudicious practice that is too frequently adopted in such cases, by the countrymen of M. *Simon*. "A young woman, of a highly nervous temperament, and subject to frequent attacks of hysteria, was seized, after a protracted and severe accouchement, with shivering, præcordial anxiety, sense of suffocation, and flying pains in almost every part of the body. The pains at length became fixed in the hypogastric region, and a profuse uterine hæmorrhage supervened. *The patient was bled several times.* The discharge became more and more abundant; and the patient was so much reduced, that great apprehensions were entertained of her recovery. Dr. *Joly*, who was now called into consultation, perceiving the utter inutility of the means that had been hitherto used, and calling to mind the *English practice* of administering Opium in many cases of uterine hæmorrhage, immediately recommended its exhibition. From this moment the patient became tranquillised, the hæmorrhage abated, and, ere long, completely ceased."—*Bulletin de Therapeutique.*

*Remarks.*—Our chief object in introducing the previous observations is to express our very decided approval of the "English practice," (as M. *Simon* calls it,) of administering Opium in cases of uterine Hæmorrhage. We almost invariably use it in our own practice. The formula, which we generally employ, is a mixture containing the Tinctures of Opium, Hyosciamus, and Digitalis in infusion of roses, given in repeated small doses. The patient should, as a matter of course, be kept very quiet, and all warm drinks be interdicted. If a tendency to the recurrence of the hæmorrhage continues, we know of nothing superior to the Sulphate of Zinc (made into pills), along with the diluted Sulphuric Acid. It is also an admirable remedy in many cases of inveterate Leucorrhea.—*Rev.*

---

### Professor Otto on the Action of different Medicines on the Mental Faculties.

All stimulant and exciting medicines increase the quantity of blood that is sent to the Brain. If this quantity exceeds a certain amount, then most of the faculties of the Mind become over-excited. Nevertheless the degree of this action is observed to vary a good deal in different cerebral organisations; and it is also found that certain stimulants exercise a peculiar and characteristic influence upon special or individual faculties. Thus Ammonia and its preparations, as well as Musk, Castor, Wine, and Ether, unquestionably enliven the Imaginative powers, and thus serve to render the mind more fertile and creative. The empyreumatic Oils are apt to induce a tendency to Melancholy, and mental hallucinations. Phosphorus acts on the instinct of propagation, and increases sexual desire: hence it has often been recommended in cases of Impotence. Iodine seems to have a somewhat analogous influence; but then it often diminishes, at the same time, the energy of the intellectual powers. Cantharides, it is well known, are a direct stimulant of the sexual organs; while Camphor tends to moderate and lull the irritability of these parts.

Of the metals, Arsenic has a tendency to induce lowness and depression of the spirits; while the preparations of Gold serve to elevate and excite them. Mercury is exceeding apt to bring on a morbid sensibility, and an inaptitude for all active occupation.

Of narcotics, Opium is found to augment the erotic propensities, as well as the general powers of the intellect, but more especially the imagination. Those who take it in excess, are, it is well known, liable to priapism. In smaller doses, it enlivens the ideas and induces various hallucinations, so that it may be truly said that, during the stupor which it induces, the mind continues to be awake while the body is asleep. In some persons, Opium excites inordinate loquacity: Dr. *Gregory* says, that this effect is observed more especially after the use of the Muriate of Morphia. He noticed this effect in numerous patients, and he then tried the experiment on himself with a similar result. He felt, he tells us, while under its operation, an invincible desire to speak, and possessed, moreover, an unusual fluency of language. Hence he recommends its use to those who may be called upon to address any public assembly, and who have not sufficient confidence in their own unassisted powers.

Other narcotics are observed to act very differently on the brain and its faculties from opium. Belladonna usually impairs the intellectual energies; Hyosciamus renders the person violent, impetuous, and ill-mannered. Conium dulls and deadens the intellect; and Digitalis is decidedly anti-aphrodisiac. Hemp will often induce an inextinguishable gaity of spirits; it enters into the composition of the intoxicating drink which the Indians call *bauss.* The use of the *Amanita Muscaria* is said to have inspired the Scandinavian warriors with a wild and ferocious courage. Tobacco acts in a very similar manner with Opium, even in those persons who are accustomed to its use: almost all smokers assert that it stimulates the powers of the Imagination.

If the psychological action of medicines were better known, medical men might be able to vary their exhibition, according to the characters and mental peculiarities of their patients. The treatment of different kinds of monomaniacal Derangement also might be much improved; and it is not improbable but that even a favourable change might be wrought on certain vicious and perverse dispositions, which unfortunately resist all attempts at reformation, whether in the way of admonition, reproof, or even of correction.—*Zeitschrift für die gesammte Medicin.*

---

### M. PIGEAUX ON DISEASES OF THE HEART.

This estimable author has recently completed his *Traité Pratique des Maladies du Coeur et des Vaisseaux*—a work which now forms a most useful compendium of information on this important branch of pathological enquiry. It is well known that M. *Pigeaux* has, for a great number of years, devoted his attention in a particular manner to the investigation of cardiac diseases. He was the first in France to impugn the accuracy of *Laennec's* hypothesis respecting the nature of the sounds of the Heart, and to shew that the mere muscular contraction of its ventricular walls is wholly insufficient to explain this auscultatory phenomenon. Although the theory, proposed as a substitute by M. *Pigeaux,* was afterwards shewn to be equally unsatisfactory as that which he had so successfully combated, no one will deny that his writings have contributed not a little to extend and improve our knowledge respecting the Physiology and Pathology of the Heart's actions.

However much disposed many persons (and we gladly rank ourselves in the number) are, in the present day, to trace back most of our valuable knowledge on medical matters to the writers of antiquity, it must be admitted that modern authors have very greatly enlarged and illuminated the domain of cardiac pathology.

It is to Mr. *Hodgson* that we owe the first comprehensive work on the diseases of the Circulatory System; and certainly the admirable notes, with which M. *Breschet* enlarged the French translation, have contributed, in no small degree, to its wide-spread popularity and usefulness. Subsequently, the writings of MM. *Dance, Bouillaud, Cruveilhier, Briquet, Velpeau,* &c., have served to explain and illustrate many obscure and difficult topics of enquiry.

To concentrate and systematise all the labours of others, and to blend with these the results of his own elaborate enquiries, has been the object of M. *Pigeaux* in the present work, and very ably has he executed his laborious task. Our author is not a mere compiler; he is a shrewd and ingenious observer. Perhaps his chief besetting sin is a love for physiological speculation. In describing the mechanism of the circulation, he attaches far too great importance, in our opinion, to the direct attraction which the capillary vessels exercise, he thinks, towards the blood. We might not object to this, if we did not find that the physiological theory formed the basis of practical deductions. But what is the case? M. *Pigeaux,* resting on the idea that the course of the arterial Circulation is mainly owing to the attraction of the Capillaries for their contents, goes so far as fondly to imagine, that the ligature in the case of wounded arteries may even be dispensed with. In perusing his writings, it is very necessary to keep this favourite dogma of our author steadily in view.

The extra-scientific appellations of *true* and *false* Aneurysm are very properly replaced by those of *traumatic* and *symptomatic* or *spontaneous.* The description given of erectile tumors, (or, as they used to be called, aneurysms by anastomosis,) is full of very interesting and instructive matter: the chapter, indeed, devoted to this subject, is one of the most important in the whole work. There is also a very valuable chapter on the diseases of the Lymphatic vessels, and another on the morbid alterations of the Blood in various diseases.—*L'Experience.*

---

### On the Diseases of Old Age.

The following remarks are from the pen of M. *Beau,* and have been derived from an extensive series of observations on the aged female inmates of the Salpétrière Hospice.

*General Pathology.*—The diseases of old age are usually of a complicated character; for, in almost every case, there is a chronic malady of some organ or another of the body, superadded to and associated with the actual and existing indisposition. It is important to bear this fact in mind, as the physician might otherwise be often apt to confound the symptoms arising from one source with those attributable to the other.

The activity of the Corporeal Sympathies being much impaired, and often almost entirely exhausted, in advanced age, it is very necessary to examine with minute attention the state of *all* the organs of the body, when any distinct and obvious disease supervenes.

The Delirium of old people differs much from that which occurs in persons of early and adult age; in the former, it is generally announced first by words and then by actions; whereas, in the latter, the reverse of this is usually the case.

Inflammation of the parotid glands is apt to occur in every form of adynamic febrile disease in old age.

Dryness of the tongue is a symptom of much greater frequency in old than in middle age. Sometimes this state exists alone, without any other appreciable sign of disease.

Shivering on the one hand, and profuse perspiration on the other, are phenomena of rare occurrence in aged patients. Subsultus tendinum also is very seldom met with in such invalids.

The acute diseases of old persons in the summer months almost always consist in affections, the basis of which is some disturbance of the Gastro-Intestinal apparatus. Such attacks may often be dissipated, almost magically, by the exhibition of an emetic and purgative. All the other most common diseases of old age, such as Catarrh, Pneumonia, various cerebral maladies, &c. are almost exclusively observed during the cold season of the year.

*Special Pathology.*—Simple gutteral Cynanche rarely occurs in old age.

Gastric disturbance is by far the most frequent disease of this period of life. Whatever be the form it assumes, the best remedy is unquestionably an emetic of ipecacuan, or, what is still better, tartrate of antimony.* Unless the attack be more complicated than usual, it will seldom resist this simple, but most efficacious, mode of treatment. As a matter of course, if any other malady be coexistent, the case will prove of more difficult treatment.

M. *Beau* has never witnessed a case of spontaneous acute Peritonitis in old age. When the chronic disease has been observed at this period of life, it has generally been the consequence of cancerous (not tuberculous) productions within the abdomen.

Laryngeal catarrh is a malady that is seldom seen; the tracheal form is of rather more frequent occurrence; but Bronchitis is infinitely more common than either. During the winter months, one half of the inmates of the Hospice are usually more or less severely affected with it.

The Catarrhal form of Phthisis—in other words, the chronic Catarrh which induces gradual consumption and death—is much more frequent than the genuine Tuberculous form in old people. During the last twelve months M. *Beau* had 9 cases of the latter malady, and 15 of the former, under his care. From this circumstance we may conclude that, whenever we meet with a general wasting of the body, frequent cough and expectoration of purulent matter in an old person, there is considerably more chance that the case is one of Catarrh than of tubercular Phthisis; and this chance is the greater, in proportion as the individual is more advanced in years. A very marked symptom of this condition is the decay, or even the entire loss, of the appetite. The emaciation of the body is probably owing quite as much to the existing Anorexia as to the purulent secre-

---

* This is a piece of excellent practical advice. The use of emetics is far too much neglected in the present day, and most practitioners are unnecessarily timid about using them in old patients. A single emetic will often effect more good in the course of a day than any other remedy in a week or more.

tion from the air-passages. M. *Beau* recommends, with the view of restoring the appetite, an emetic in the first instance, and then the decoction of Polygala or of Lichen islandicus, Morton's balsamic pills, syrup of Tolu, Bourdeaux or Bagnols wine. Fortunately this medication generally serves a double purpose; for it exerts a beneficial effect on the condition of the respiratory as well as that of the gastric apparatus. In three cases of this Catarrhal phthisis, which proved fatal, our author could not discover on dissection any traces of decided lesion in the mucous membrane of the bronchi.

The course of genuine tuberculous Phthisis is generally very slow in old age; there is seldom any colliquative sweats or purging. In most cases, the deposition of the tuberculous matter is limited to one or two circumscribed parts of the upper lobes, and is not disseminated through the substance of the lungs. On one occasion only, were tubercles found in both lungs.

M. *Beau* is inclined to adopt M. *Laennec's* opinion respecting the spots of induration not unfrequently met with on the pulmonic surface, and to regard them as produced by the Healing or cicatrisation of small tuberculous deposits. He comes to this conclusion, 1, because he deems it very unlikely that these indurations are produced either by apoplectic ecchymosis, purulent formation, or circumscribed infiltration of the pulmonic substance; and 2, because, out of 176 non-phthisical aged women, in whose cases he had made a post-mortem examination, he found Cicatrices on the upper lobes of the lungs in no fewer than 173 cases. According to the researches of M. *Louis*, tubercular phthisis is of more frequent occurrence in the female than in the male sex; and the accuracy of this remark is borne out by the experience of M. *Beau* in reference to aged people.

Pneumonia in old age very often presents an Intermittent character, under the influence of treatment; all the symptoms, physical as well as rational, sometimes ceasing (after a bleeding for example) for an entire day or so, to return perhaps on the following one with its accustomed or perhaps aggravated severity. This temporary *lull* of the symptoms may occur three or four times, in the course of the treatment. Hence the necessity of the physician being very guarded in his Prognosis as to the ultimate issue, and of his being cautious even in giving an opinion respecting the probable duration of the disease; for a patient may seem to be one day on the fair road to recovery, and, the very next, his case may turn out to be utterly hopeless.—*Journal de Medecine.*

---

### Non-injurious Effects of Solitary Imprisonment with Labour.

The recently-published reports of the state of the prisoners in the *Roquette* prison at Paris, in reference to their bodily and mental health, quite confirm the statements that have been made on this subject by the physician and other officers of the United States prisons, at Cherry Hill and Moyamensing in Pennsylvania. The system pursued is constant Solitary Confinement in separate cells, in conjunction, however, with regular occupation, exercise in the open air, and permitted visits occasionally from relatives and other persons. Each cell is thus made to become a special and complete Prison in itself; and the prisoners are prevented from having any communication whatsoever with each other. Their attention is kept constantly engaged in some useful and profitable employment; and moreover they hear and see nothing that can in any way remind them of their vicious pursuits. It has however been supposed that, even under these advantages, Solitary Confinement is apt to bring on, in many cases, a more or less confirmed degree of insanity.

But this idea seems to be scarcely borne out by well-established facts. The medical officer of the great American prison at Cherry Hill gave his evidence on this subject, in the following terms: "You ask me if solitary imprisonment with work has seemed to me to encourage a tendency to Insanity. So far as I have been able to judge, I feel strongly convinced that this plan, so far from being injurious to the general health of the prisoners, has been decidedly beneficial in every respect, alike to mind and body. Since indeed the prisoners have been confined in the new prison of the county, I have met with a good many cases of Mental Derangement; but in no single instance have I had reason to believe that the regime of the prison had anything to do with producing the malady. So far from this being the case, I am rather inclined to assert that the solitary confinement—in conjunction (be it always remembered) with regular labour and bodily exercise—has materially contributed to the recovery of the reason, in a good many instances."

From the official reports, that were submitted to the Senate of Pennsylvania, we learn that the mortality in the prison, during last year, did not exceed that of the free population in the city; and moreover, that not a single case of Insanity occurred during the whole twelve months—although no fewer than 26 cases were met with in the year 1839.

So much for the state of the Pennsylvania prisons. In the *Roquette* prison at Paris—which has now been established for upwards of four years, and in which from four to five hundred young criminals are usually confined—not a single case of Insanity has occurred among the prisoners; and yet one might, *à priori*, have supposed that solitary confinement should have been more injurious, in this respect, in youth than in adult age. As to the ratio of the Mortality, this has not exceeded from 7 to 8 per cent.—being not above one-half of the average mortality among the lower classes of the inhabitants in the French metropolis. In his report for last year, the Prefect of the police says:—"The robust population of the prison—who, before the regime of solitary *encellulement* was introduced, were thin, pale, and ill-looking—have, for a length of time past, exhibited a very marked change for the better, in their general appearance; they look healthy, and not a few of them appear to be cheerful and contented too. The fears which we, in common with many other individuals, entertained as to the effects of the Solitary System on the youthful prisoners in the *Roquette*, have been completely dissipated by the results which we have witnessed during the last two years."—*Annal. Med. Psych.*

---

### NEW EDITION OF THE WORK OF CABANIS.

It is always interesting, and withal often very instructive, to look back at some of those writings, which, in the life of our fathers, and in the old time before them, exercised a wide influence upon public opinion. The period, that immediately preceded the outbreak of the French revolution, must always be regarded by the thoughtful reader as one of especial interest in its bearings on literature as well as on the higher interests of politics, morality, and, above all, of religion. Few of the works of that date were received with more eclat at the time, or are more likely to retain a place in the library of the student, than the famous 'Rapports du Physique et du Moral de l'Homme.' Dr. *Cerise* has recently brought out a new edition of this celebrated Physiological and Psychological book; appending to it not only a life of the author, but (what is more valuable) numerous annotations and comments, with the view of exposing the fallacy of many of its arguments, and the dangerous tendency of its general doctrines. The following notice of this new edition—published by Fortin and Masson of Paris—is taken from the pages of the Revue Medicale.

"The name of *Cabanis* is unquestionably one of those which best represent that famous philosophic School that flourished towards the close of last century, and whose aim and object seemed to be to establish and diffuse, among us, the principles of Materialism. In spite of some isolated phrases and expressions, in which the author protests—but most unsatisfactorily—against this alleged tendency of his writings, it must now be acknowledged by every unprejudiced reader that they form a constituent portion of that Literature, which exercised, for a length of time, so pernicious an influence on the morals and general social character of our countrymen. The author has indeed, with great ingenuity, developed his thoughts on the intricate question as to the mutual relations between Mind and Body, illustrating it in a variety of ways, and rendering it withal as attractive as possible by the graces of an accomplished scholarship. Representing most faithfully the doctrines of the Encyclopedic philosophy—the starting-point, we may remark *en passant,* of the writings of *Broussais* and several of his disciples—the 'Rapports, &c.' had a prodigious success at the time of its publication, a success which it has in some measure retained even to the present time. Every medical library, now-a-days, must have its copy of *Cabanis;* and what author is there who pretends to treat of the relations of the physical and moral constitution of Man, that can dispense with quoting the standard work on the subject?—whether it be with the aim of impugning some of its doctrines, or of merely parading them before the eyes of the multitude in the way of illustration and argument. Public opinion has at times exalted this work so high, as to make it quite a standard of doctrine, and one for the possession of which the most animated controversies have been sustained.

"Dr. *Carise,* as the editor of Cabanis, has not, we rejoice to say, been contented with following the usual practice of writers under such circumstances; to wit—acting as the mere Panegyrist of his author; he has imposed on himself a far higher and more arduous duty, and one for the performance of which the religious public have much cause to be grateful to him. While ready on all occasions to do ample justice to the doctrines enunciated by his author, he does not fail, whenever a becoming opportunity presents itself, to show how incomplete, and often how utterly erroneous they are. The fallacy and dangerous tendency of much of the reasoning of this popular work are thus effectually neutralised, so that the student of the present edition is no longer apt to be led astray by the Casuistry and false teaching of the original. He points out most perspicuously and with great force how necessary it is, in all metaphysical investigations of this kind, to have a clear and distinct notion as to the meaning of the Terms, the *moral* and the *physical constitution* of man; and, after shewing the objections to which these are liable, he proposes to replace them with the simpler ones of *organism* and *idea.* The first he defines to be 'the *ensemble* of those organic phenomena which, by not being associated with any idea, are produced without our cognizance;' and the second, as 'the *ensemble* of those organic phenomena which, by being associated with an idea, are cognizable by our minds.' This preliminary step being settled, it is comparatively easy to determine the definition that may be given of that Branch of physiological science which professes to investigate the mutual relations between the corporeal and the moral constitutions of our nature:—it is nothing more nor less than 'the co-ordination of the relations, in virtue of which the Organism and the Idea mutually act and re-act upon each other.'

"Some writers have fondly sought to represent the Organic Operations of all living bodies—those, we mean, which are common to the animal and vegetable kingdoms,—as the evidences or manifestations of a Thinking Principle: such men belong to the school of the *pantheists.* Others represent the intellectual and moral faculties of man—to which there is certainly nothing analogous in the lower animals—as the mere manifestations of the Vital Properties: these are the *materialists.* It will be observed that both of these strange doctrines equally deny the duality, so to speak, of the human constitution; for, while the one asserts that everything is mere Matter, the other seeks to show that everything is mere Spirit.

"Dr. *Cerise* very happily exposes the delusive tendency of both these views, and he shews that the same mode of reasoning, when applied to the case of physiological pursuits, inevitably leads the enquirer into the same errors. For example, it is well known that *Stahl* regarded the intelligent soul as the supreme Directress of all the organic and moral phenomena of our nature. *Cabanis* and his disciple *Broussais*, took the very opposite view of the question, and considered the material organism of our bodies as the Source and spring of all the faculties and feelings of the mind.

"May it not be asked, how comes it that *Cabanis* should strive to found a science, which in reality he laboured to destroy, when he affirmed the material Unity of man? and how did he fail to recognise that obvious Duality of the human constitution, without the admission of which, the science, of which he professes to treat, can have no existence? In truth, the blindness of the author's mind on these points was owing much more to the disturbing influence of pre-conceived and pre-adopted notions, than to any ignorance on that subject, which he so ingeniously discussed: the cause of the error was seated in the *heart* rather than in the *head*. And where is the man who could ever calmly and impartially investigate any intricate subject of Ethical metaphysics, when his mind was tainted with the pollutions of a loose morality or of a scoffing infidelity? The thing is impossible. As the spring is, so will the water of the fountain be; if the tree be corrupt, the fruit and the branches must be corrupt also. To point out the grave error above alluded to, in the work of *Cabanis*, has been the aim of his learned Editor; and well has the latter gentleman performed his task: the shaft is no longer poisoned; nay, it rather carries an antidote to the very wound which it serves to inflict.

"In conclusion, we may safely say, that Dr. *Cerise* is entitled to very high praise for the manner, in which he has performed his duty as the Editor of a work that has been, and is likely still to be, very widely known. He has taught us its real value, and, without depreciating its unquestioned merits, he has reduced its authority to a legitimate standard.

"Henceforth the reading of the 'Rapports, &c.' can no longer be regarded as dangerous to the young student of physiology; for certainly there is little chance of his being seduced by the Materialist conclusions of the author, if he will but peruse with due attention the admirable prefatory remarks of the present edition. —*Revue Medicale.*

---

### A Nosological Text-book for the Medical School in Egypt.

During the course of the past year, there was published in Paris a volume entitled "Cours de Nosologie Clinique, par Dr. *Emangard*," professor of pathology and clinical medicine in the medical school at Cairo. This work has been translated into the Arabic language, by the express command of *Mehemet Ali*, for the use of the native students. Unfortunately it is thoroughly pervaded with the spirit of the Broussaian doctrines. Every disease seems to be, according to the author, an Inflammation and nothing else. There is not a chapter or section of the work, in which we do not meet with an ever-recurring mention of the universal phantom, *gastro-enterite*. What, for example, is Gout?—answer, a mere chronic inflammation of the stomach. What is Typhus fever?—a gastro-enterite. Yellow fever?—the same. Cholera-morbus?—aye the same. And even the Plague, the in-dwelling pestilence of the country, is set down in the same category of the nosological scheme.* The mother-idea of *Broussais* is transparent in every part of this work. Unfortunately Dr. *Emangard* is a devoted disciple of the grand reformer, not only in theory but in practice: he is, to say the least of it, consistent; what he teaches in the closet he practises at the bed-side; what he declares from the professor's seat, he enforces in the wards of the hospital. The treatment of diseases, a subject that is so difficult for physicians who have not the *clair-voyance* of those who proclaim themselves to be the only true Physiologists, becomes in his hands the most simple matter in the world. Bloodletting and low diet constitute the principal, we should rather say the only, basis of anti-irritative therapeutics. We may notice one point of difference between the system of treatment recommended by the Master and that pursued by the Disciple: the former invariably ordered gummed water, while the latter uses nothing but the pure unadulterated element!

Having settled the etiology of all the genuine Pyrexiæ and Phlegmasiæ, our single-idea author proceeds to describe the genuine Neuroses—which, according to his nosological creed, are, as a matter of course, all referrible to mere irritation in some part or another. He founds his belief on this subject, he says, on the results of those decisive (to him) experiments which have established the perfect identity of the Nervous and the Electric agencies; and, proceeding from this starting-point, he endeavours to shew that every Neurosis is due to an accumulation of Electricity, just as every Phlegmasia is due to the accumulation of Blood, in the affected part or organ of the body. He adopts to the full extent the idea of *Broussais,* that the nervous power or influence is truly and strictly one form of Matter—which therefore may, under certain circumstances, be present either in an excessive or in a deficient degree throughout the system. Once that this point is determined—viz. the materiality of the nervous power—Dr. *Emangard* finds, as may be readily supposed, no difficulty in explaining the nature and proximate cause of the Neuroses: they are placed on the same line as the Phlegmasiæ, and constitute one of the two species which form the *genus* 'Irritation.' He seems to regard the human body as a mere large and complex Electrical Machine, which, by becoming charged with varying proportions of the vivifying fluid, exhibits the conditions of excessive or deficient innervation. All this is vastly ingenious: but the important question comes to be, is it true? The first-year medical student can answer the question as well as ourselves.—*Revue Medicale.*

---

### Variations in the Climate of France.

Every classic reader knows full well how severe the Winters used to be in France during the time of Julius Cæsar, when even the Rhone was frequently so firmly frozen that troops, with all their baggage, could be passed over it without difficulty. A vast portion of the country was then covered with dense Forests, which, by excluding the rays of the sun, and by retaining a continual cold damp fog on the surface of the ground, must always contribute very powerfully to the reduction of the general temperature. There is every reason to believe that, in those days, the Vine and Fig-tree were scarcely, if at all, known in France.

The climate seems to have become more genial in the course of the first Christian century. M. *Fuster,* in his recent memoir read before the Academy, very clearly

---

* We need scarcely add, that Dr. *Emangard* is a most strenuous anti-contagionist. He utterly rejects everything that is at all opposed to his view of the question, and, as a matter of course, inveighs very forcibly against the inutility of all Quarantine establishments.

proves the truth of this, by tracing the ascensional advance of the cultivation of the vine, from the South towards the North.

Before the time of *Strabo* arrested, so to speak, at the foot of the Cevennes, it (the vine) now began to pass over this barrier : *Columella* found it in the country of the Allobrogi (Dauphiny), and *Pliny* saw it growing spontaneously in Auvergne and even in Franche-Comté. In the year 69 of the Christian Æra, when the Emperor *Domitian* ordered all the vines in France to be destroyed, we learn from history that the cultivation of this plant had not been carried farther North than the environs of Autun. Subsequently however, in the reign of *Probus*, we find that it was cultivated along the banks of the Seine, and also that the fig-tree followed, but at one or two degrees further South, in its wake. *Julian*, who lived for a short time in the small town of Lutetia (Paris) during the 4th Century, has drawn a charming picture of the surrounding district. He praises the great mildness of the climate, the excellence of its vines, and the rapid multiplication of its fig-trees. We learn also, by one of his letters, that the corn was quite ripe, at the Summer Solstice, even in the northern provinces. During the course of the following century, the climate of France appears to have become still warmer and its soil more fertile; for even Normandy, Picardy, and Brittany then produced abundant harvests of grapes, and the wine thus procured was not a little esteemed. The vintage usually took place in September, but some years as early as August. The corn harvest was very generally in the first or second week of July. This genial state of things seems to have continued for several centuries; and it was not until the 12th or 13th century that any decided signs of deterioration of climate were anywhere remarked. One of the Norman *fabliaux*, of the time of Philip Augustus, expressly mentions the wine of Beauvais as one of the very best in the kingdom; but even at this period (1228—1239), it seems that the vineyards had very sensibly decayed in the most northern districts, and that in the neighbourhood of Cherbourg they had nearly quite disappeared. The district of Eu was then famous for its apples, and for the fine cider which the inhabitants made. As the vineyards in different parts failed, the orchards gradually increased; so that, in course of time, almost the entire extent of the northern provinces—including Flanders, Artois, Normandy, Brittany and Picardy—became and has remained an apple-growing country, the vine thriving only in a few sheltered spots.

The alteration in the climate of France was at first confined to the Northern Provinces; it reached the Southern ones only step by step, and at a considerably later period. The vines of the neighbourhood of Saon had a very high repute at the close of the 15th Century; and many of the writers of the following century celebrate the richness and strength of the wines that were still made in the environs of Paris, especially those of Argenteuil, Marly, and Montmartre. The vintage was then in the month of September. At this time too, orange, lemon and citron trees flourished without difficulty throughout the whole of Provence, and in many districts of Languedoc ; and even the sugar-cane, we are told by *Ollivier de Serres*, was fairly acclimated in the former province.

Our climate, adds M. *Fuster*, continued very sensibly to deteriorate from the North to the South, during the 17th and 18th Centuries. The Paris wines fell into discredit; the orange and lemon trees no longer thrived in Languedoc; the sugar-cane entirely disappeared from Provence; and the olive tree gradually receded to the Southern Coasts. Notwithstanding, however, these changes, the central and meridional districts of the kingdom still retained a warm and very genial climate. The wines of Orleans were esteemed among the best in the country during the 17th Century; the olive tree flourished at Carcassone, and along a great portion of the East Coast; palm trees were abundant in Provence, and the whole champagne district of this province between Orgon, Aix, and Marseilles, produced large quantities of oranges and lemons, as fine as did the country between Marseilles, Hyeres, and Frejus.

The 18th Century deprived our climate of all these advantages. It has witnessed the last vintages of Normandy and Brittany; the impoverishment of the vineyards in Maine, Anjou, and Orleans; the retreat of the olive beyond Carcassone, and of the orange still farther South; and it has reduced the palm-trees of Provence to a state of entire unfruitfulness. During the last 60 years, the deterioration of the climate of France appears to have been continually going on. In the present day, the grape does not ripen very readily in the open air in the northern provinces; even many of the finer stone-fruits, such as the peach, nectarine, &c., do not thrive there at all well, except on espalier trees; and the olive no longer grows at Carcassone, nor to the North of Montelimart, on the left bank of the Rhone. *Decandolle,* in 1835, calculated the retrogradation of this tree in the department of the Aude at 5 *myriametres,* since the year 1789. It appears also, from the researches of Malte-Brun, that in the present day the wheat of France is less productive, by nearly one-quarter, than it was in that year.—*Gazette Medicale.*

*Remarks.*—The facts mentioned by M. *Fuster,* respecting the changes in the climate of France, are not undeserving of the attention of the medical philosopher, as we need scarcely say that the character of the Endemic, as well as of many of the Occasional and accidental Diseases of a country will always be very materially affected by any great alterations in its atmospheric and terrestrial conditions. We were not aware that the average temperature of France had so very sensibly declined within the last century or two, as it seems from the researches of our author to have done.

---

### M. Donné on the Microscopic Examination of the Fluids.

The recently-published work—Cours de Microscopie—of this gentleman is, as might be expected, attracting much notice among his professional brethren in his own country. It is a most elaborate performance, and evinces the zeal and ability of the author in discussing his favourite subject. All the Fluids of the body, from the Blood to the Synovia of the joints and the humours of the eye, are successively brought under review, and their microscopic and chemical characters described with great exactness. The following brief *excerpta,* from some of the chapters, will enable our readers to judge of the general style of their contents.

The formation of the Buffy Coat depends, according to his observations, on the density of the blood and the time it takes to coagulate; the richer it is in solid matter, the more time is required for its perfect coagulation. The *maximum* of density, however, seems rather to impede, in a certain degree, this process: hence the buffy coat is often much stronger at the *second* than at the *first* bleeding, in many cases of Acute Rheumatism and Pneumonia. The degree of density appears to be commensurate with the quantity of the albumen, rather than of the fibrine, existing in the blood. When very dense, the Coagulation occupies at least 12 or 15 minutes, before the buffy coat is fully formed. When the coagulation is very rapid, as is usually the case in Typhoid fever, (in which disease the blood will sometimes gelatinise in three or four minutes,) there is not time enough for the formation of any buffy coat. As a general remark, it may be stated that there is seldom a distinct Buffiness formed, when the process of Coagulation occupies less than ten minutes. M. *Donné* draws, from the coagulation of the blood in the blood-vessels, an ingenious sign to determine the presence of Death. When life is extinct, the blood, received into a watch-glass, will be found to produce only a reddish serosity, but no clot; for this, already coagulated, remains behind in the blood-vessels.

In his elaborate description of the red Globules, our author expressly denies the commonly-received assertion of many physiologists that these bodies are *nucleated*. In the blood of the adult at least, there are certainly no nuclei present in the globules; but it seems probable that they may exist in those of the young embryo. He alludes particularly to the fact that the globules, after the escape of the blood from the vessels, are sometimes apt to become clustered together; they then form chaplets, or bead-like rows, &c. Not unfrequently some of them have a crisped appearance, and are less transparent than the others: this has been considered, but incorrectly, to be the result of a diseased condition. There is no difference, according to our author, between the globules of Venous and those of Arterial blood.

In reference to the blood in the embryotic chick, M. *Donné* says that the globules are distinctly visible before any appearance of the Heart can be discovered, and that they are already subject to an oscillatory movement in the direction from the periphery to the centre, where the future heart is situated. This opinion, although in accordance with that professed by most physiologists in the present day, is, according to Dr. *Lebert*, the reviewer of Dr. *Donné's* work in the Annales de Chirurgie, not correct. His researches, carried on along with Dr. *Prevost* of Geneva, have led him to the very opposite conclusion; viz. that, in the chick, the heart and vessels are formed before the blood-globules. These, he says, are developed in the translucent fluid already contained within vascular parietes, and then the earliest movement communicated to the columns of the blood begins at the Heart, and has a centrifugal character—a character which however seems to be oscillatory during the first hours of the circulation, because the contractions of the heart have not yet the due degree of power, which they afterwards possess.

This section of the work is closed with a very interesting observation on the subject of Transfusion; to wit, that the accidents, consecutive on this operation, are attributable not to the different form of the Globules of the injected blood, but rather to the coagulation of its Fibrine; and that any and every sort of blood may be injected, in certain quantities, into the veins of an animal without producing any alarming consequences, provided the fibrine has been previously removed.

M. *Donné* then proceeds to describe the white or colourless globules, and also what he calls the Globulines of the blood. The former resemble the globules of mucus; they contain three or four solid granules within; they are not affected by water, but are condensed and contracted by acetic acid. In the process of coagulation, they will be found present in a greyish stratum between the serum and the red layer of the globules.

By the term 'globulines,' our author designates the minute granules, observable in a state of molecular movement in the blood. He is of opinion that the white globules are the globulines of the Chyle, uniting with each other in groupes of three or four, and becoming enveloped with an albuminous covering, by rolling about in the sanguineous fluid. But this is still very uncertain. In the embryo of the Batrachian animals, we have been able to watch the primitive organoplastic globules becoming transformed into blood-globules.

A part of the Globules becomes dissolved in the liquid portion of the blood, transudes through the walls of the capillary vessels, and thus constitutes the nutritious and Organizing Fluid of the body. We have been able, on several occasions, fully to satisfy ourselves on this point; to wit, the Solution of the globules in the liquid part of the blood; for, in our experiments on the process of inflammation, we have distinctly seen the fluid, which surrounded the irritated and engorged vessels, become tinged with a red colouring matter: this could proceed only from dissolved globules, as there were none visible on the outside of the vessels.

To shew the Circulation of the blood to his pupils, M. *Donné* has been, for some time past, in the habit of employing the tongue of the frog, as the object under the microscope—an ingenious idea; for the mechanism of muscular contraction can be witnessed at the same time. The play of the circulation is very expressively compared to a geographical map, in which all the rivers and streamlets are supposed to become suddenly animated. The rapid and whirling-like circulation around the mucous follicles is also observable, at the same time.

In his description of the morbid alterations of the blood, M. *Donné* first gives a succinct history of the late admirable researches of MM. *Andral* and *Gavarret*, and he then details the results of his own observations in this interesting department of pathological enquiry. We shall briefly notice a few of these. In Chlorosis, the red-globules are *decolorés*, and more transparent than in health; in Typhus Fever, they are not at all changed from the normal standard. The globules of purulent matter are not distinguishable in the blood from the white globules, which normally exist in it. These latter may, under certain unknown circumstances, exist much more abundantly than they do in a state of health. This anomaly is present more particularly in the blood of patients, who have been long labouring under some grave disease or lesion, and whose systems are enfeebled by protracted illness.

The blood sometimes exhibits an oily condition; and its globules, instead of being separate and distinct, are agglomerated together in chaplets or little groupes. The occasional white or milky appearance of the blood is owing to the admixture of untransformed chyle with the circulating fluid. M. *Donné* relates a curious example of this anomaly.

*Mucus.*—This secretion is found, on microscopic examination, to be composed of a viscid stringy fluid, and of a solid matter that consists chiefly of shreds of the epithelium. It is sometimes acid, and at other times alkaline. M. *Donné* distinguishes three kinds of mucous membranes: 1. Those that are analogous to the skin, which furnish a frothy acid secretion; for example, the lining of the vagina. These acid-mucous membranes, which our author calls *false*, never exhibit any vibratory cilia on their surface. 2. *True* mucous membranes—as that of the bronchi—which secrete a fluid that possesses alkaline properties, is viscid, and contains mucous globules: these are supplied with vibratory cilia. 3. Intermediate mucous membranes, which secrete a mixed kind of mucus: of this kind are those which exist around the orifices of the mouth, nose, anus, &c.

The globules of the *true* mucus, as that secreted by the air-passages, are, in our author's opinion, very nearly identical with those of purulent matter. He therefore seems inclined to attach but little value, in diagnostic enquiries, to the mere presence of pus in the sputa, and thinks that no very trustworthy inference can be deduced from this phenomenon as to the gravity of an existing disease. M. *Donné* has probably carried his scepticism on this point somewhat too far, although we are quite disposed to agree with him, that we must not attach too much importance to the microscopic appearance of the sputa in suspected tuberculous disease of the lungs; as we have more than once failed to detect any traces of tuberculous matter in the expectoration, even when the tubercles had unquestionably become softened, and vomicæ had formed. In the mucus of the alimentary canal, we observe numerous altered modified globules, and conical epithelial cells; that of the vagina contains many epidermic vesicles and minute filaments. Our author has discovered in the vaginal mucus a very curious infusory animalcule, which he calls *Tricho-monas Vaginæ.*

As we have already said, M. *Donné* denies, and justly so, the possibility of distinguishing pus from mucus by the microscopic characters of their globules alone; and he shews that it is rather in the fluid than in the solid constituents of these secretions that any distinctive features are to be sought for. In the case of the former fluid, the globules are observed to roll about in the serosity in which they float; whereas, in the latter, they are much more sluggish and quiescent, so to speak, in consequence of the viscidity of the medium in which they are suspended. Dr. *Lebert* does not entirely agree with our author as to the alleged resemblance, far less as to the identity, of the globules of mucus with those of pus. It requires however, he admits, a much higher magnifying power than is usually employed to detect, with any degree of satisfaction, the differences that really do exist. There is one fact, insisted upon by M. *Donné*, that deserves especial notice here: to wit, that the admixture of pus with fresh-drawn fluid blood renders the fibrine of the latter soft and diffluent; the coagulum which then forms, retaining very little consistence, and becoming of a dark livid hue: part also of the colouring matter becomes dissolved in the serum.

Purulent matter often contains *Vibrios;* but, as these Animalcules are so frequently found present in other animal fluids, we are forced to regard them as a mere product of decomposition.

In the Sixth Chapter, M. *Donné* gives a very interesting description of the Vibratory Cilia, which exist on various mucous surfaces. The Epithelium consists of a series of imbricated cones, the free extremities of which are provided with vibratile Cilia; these, by being in continual motion, serve most effectually to promote the course of any fluid which may be present on their surface. After a certain time, the vibratile Epithelial Cellules become detached from each other, and float about free in the fluid. According to our author, they possess in this state a power of spontaneous motion; he compares them to the Spermatic Animalcules.

In the Seventh Chapter, the characteristic phenomena of most of the Secretions, such as the sweat, the saliva, the bile, urine, &c. are described at great length. The matter of Sweat has no proper microscopic particles; it generally exhibits acid properties; but is alkaline in some parts, as in the arm-pits, between the toes, and around the organs of generation. The Saliva is in itself alkaline; but, from being mixed with more or less of the mucus of the mouth, it usually exhibits an acid reaction. When evaporated, it presents very beautiful crystallisations, which recal the forms of Sal Ammoniac. The Bile is an alkaline fluid; it does not exhibit any well-marked particles under the microscope. Its presence is easily recognised in any fluid by the re-action of Nitric Acid—which turns it successively to a deep green, then a blue, and lastly a red colour, if we continue to add the Acid by degrees. The microscope is, therefore, of great value in detecting the presence of Bile, even when it exists in very small quantities indeed.

Passing over the inorganic constituents of the Urine, we may briefly enumerate the organised sediments which are not unfrequently found present in this fluid. 1. White filaments, which consist of mucous globules, epithelial scales, and occasionally also of spermatic animalcules: these substances usually come from the canals of the prostate gland. The cloud, that is often to be seen in healthy urine, is also composed of Epithelial scales and of Mucous matter. 2. The Mucous deposit properly so called; it has a greyish semi-transparent appearance, and contains globules of mucus held together by a stringy matter, and also scales of Epithelium. 3. Purulent matter: this is usually seen in a circumscribed layer of a dull opaque whitish aspect. 4. Blood, recognisable by its peculiar globules, which are soluble in Acetic Acid and in Ammonia, but are insoluble in Nitric Acid. 5. Spermatic fluid: this is always present, in greater or less quantities, in urine voided immediately after a seminal emission. 6. A peculiar fatty matter, which separates on the cooling of the urine: it communicates a whitish troubled appearance to the urine, which however becomes transparent on the addition of Ether. The pathognomonic value of this phenomenon has not hitherto been accurately determined. 7. Chylous urine: this always contains a certain quantity of blood which settles down to the bottom of the vessel, while its surface is covered with a pellicle of a creamy-looking matter. This peculiar state of the urine is of frequent occurrence in some tropical countries. The serum of the blood too, in such cases, has usually a milky appearance.

The preceding remarks as to the organic sediments in the urine, apply chiefly to this secretion, when it exhibits acid properties; but nearly the same deposits are observed in alkaline urine also—only that, in the latter case, the globules are more disintegrated and reduced to the state of globulines; they are generally transformed into a glairy stringy substance; and the Zoosperms are usually more or less completely destroyed.

With respect to these *Animalcules*—of the *Semen*—our author thus expresses his opinion respecting their Animality:

"We perceive, by considering their mode of Generation, that they seem to be rather *animated particles* which are detached from a living organ, than animal existences properly so called." They may be viewed as the product or result of a secretion, as the vibratile Epithelium is; nevertheless, they certainly possess in a much higher degree than it the power of spontaneous motion. * * * * They quickly die in the mucus of the Vagina when this has become acid, or when a woman is pregnant; also in that of the Uterus, when it contains an excess of alkali. *Leuwenhoeck* detected the existence of Zoosperms in the Uterus and Fallopian tubes—a discovery, the truth of which has been amply confirmed by the recent observations of *Bischoff* and *Barry*. From what we have now said, it may be fairly concluded that the vitiated secretions of the generative organs may be a frequent cause of Sterility. Leucorrhœa has not this noxious effect to the extent that has generally been imagined; it is only when the mucus of the Vagina has become too acid, or contains an excess of alkali, that this result may be expected. M. *Donné* devotes a chapter to the consideration of morbid Seminal Emissions: he regrets, as altogether unsatisfactory, the explanations which have been usually given as to their etiology, and he expresses his entire disapprobation of the treatment of the complaint by the application of any caustic to the urethra. The best remedy, in his opinion, is the copious and steady use of cold water, both internally and externally.

After giving a minute account of the microscopic characters of Milk, our author describes at considerable length the peculiar constitution of the *Colostrum*. It is a yellowish and viscid fluid, but without having any predominance of the butyraceous constituent. Under the microscope it exhibits, besides imperfectly-formed globules of milk, numerous granules, which appear to be surrounded with an envelope, and sometimes contain within their own substance a few globules of milk. There is (so some have thought) considerable analogy between the globules of the Colostrum, and the large granular globules, which form one of the earliest and most frequent elements of inflammatory exudation; let us remember that, in truth, the first step in the secretion of milk is evidently accompanied with a highly congested and hyperæmic state of the Mammæ, which receive a large portion of the blood, that was previously directed to the uterus, for the nutrition of the fœtus. Moreover, these corpuscles of the Colostrum are found in the milk, whenever the Mammary gland becomes the seat of inflammatory or suppurative action; and also during those Epizootic diseases which are attended with these two orders of phenomena.

The engorgement of the breast produces granular and mucous (purulent) globules—a circumstance which confirms the view we have taken of the Colostrum. Pus is occasionally found blended with the milk: purulent milk constitutes, in the lower animals, the Epizootic disease that is known by the name of 'cocotte.' M. *Donné* has discovered globules of pus in the milk of cows affected with phthisical disease of the lungs. The admixture of milk with the blood is of rare occurrence in women, but more frequent in the lower animals. If the Catamenia are present during lactation, a number of anomalous granular corpuscles have been observed in the milk.

Of the various means that have been proposed to preserve milk, M. *Donné* gives the preference to ice used in an apparatus, which consists of two hollow cylinders, the ice being put into the inner and the milk into the outer one: the ice may require renewal every 12 hours or so.

The closing chapter of the work describes with accuracy the microscopic characters of the Chyle, the Lymph, the Synovia, the Vaccine virus, &c. The last-mentioned fluid exhibits, we are told, no specific characters, even under a tolerably high magnifying power.

Among his observations on the humours of the Eye, our author remarks that the *Muscæ volitantes,* the spider-web, &c.—which many persons see floating before their eyes, and which are in general composed of globules that are also seen isolated—must be referred to the anterior Capsule of the Lens, to the humour of *Morgagni,* and to the substance of the lens itself.

These extracts will suffice, we should think, to shew the general character of M. *Donné's* work, the variety of subjects that are discussed, and the ability with which they are treated.

---

### THE FUNCTIONS OF THE ACCESSORY NERVE.

*Goerres,* as far back as 1805, suggested the idea that the Nervus Vagus constituted the posterior root, and the Accessory Nerve of *Willis* the anterior root, of one of the Spinal nerves. This opinion was adopted by *Scarpa;* and its accuracy has been subsequently confirmed by the experiments of *Bischoff, Arnold, Longet,* &c.

In the well-known work of *Wrisberg* on the Nerves of the Pharynx, we meet with the following note: "Ex vulnere, quod musculum Trapezium illo in loco penetravit ubi nervus Accessarius per eundem distribuitur, ab ipso læsionis momento loquendi difficultas enata, et per reliquam vitam superstitem in balbutiem mutata fuerit."

From the reseaches of the physiologists now quoted, we may fairly conclude that the Nervus Vagus is a nerve of Sensation, and the Accessory nerve is one of Motion. The numerous experiments, recently performed by M. *Morganti,* abundantly establish the truth of this conclusion. In every case, where he laid bare and irritated the Accessory nerve—whether in the Vertebral canal between the Atlas and the Occipital bone, or on the exterior of this—he found that contraction of the Trapezius and Sterno-mastoid muscles was induced; while the animal did not exhibit any symptoms of pain. When he divided the nerve at its point of entrance into the *foramen lacerum,* the voice was observed to become immediately rough and hoarse, and to have a sort of whistling and hissing noise—the result of the vocal chords being paralysed.

The following are the conclusions which M. *Morganti* draws from his researches:—

1. The Accessory nerve is a nerve of Motion.
2. By its external branch, it is a motor of the muscles on which it is distributed.
3. By its internal branch, it is a motor of the intrinsic muscles of the Larynx: it is therefore the nerve of the Voice.
4. The external branch is formed by the filaments, which arise first from the Spinal Marrow; in other words, by the inferior filaments.
5. The internal branch is formed by the last filaments; that is to say, by those which arise below the Nervus Vagus. This branch in part supplies the pharyngeal nerve; it constitutes the recurrent nerve; and it gives off various motor filaments along its course or *trajet.*

6. The Accessory nerve constitutes the anterior root of the Pneumogastric nerve.—*Annali Universali; and L'Experience.*

Strange! that the subject of motory and sensory nerves should be discussed, without even any mention of *him* who first elucidated and explained its intricacies. As well might our continental confreres seek to omit the name of *Watt* in connection with the Steam-engine, or of *Faraday* with the science of Electricity, as that of *Charles Bell* with the Physiology of the Nervous System.

---

### Anatomy of the Chinese.

M. *Liautaud* communicated last January to the French Academy an Anatomical Engraving of the viscera of the head and trunk, which had been sold by a medical man of *Tching-hai,* after the recent capture of this place by the English troops. The date of this engraving is 1576—a period at which the Chinese could not certainly have had any knowledge as to the state of anatomical information in Europe. The letter-press, which accompanies it, is entitled a Description of the Organs of the Human Body. These are divided into the *tsang,* —which correspond to our parenchymatous viscera, and comprehend the Lungs, Heart, Liver, Spleen and Kidney—and the *fou,* or the Membranous Viscera, including the Stomach, the large and small Intestines, the Urinary and Gall Bladders. The Brain also is represented: it is called the *mother of the spinal marrow,* which is prolonged down to the os coccygis. The Trachea, on reaching the chest, is made to divide into no fewer than seven tubes, of which four proceed to the left lung, two to the right, and an intermediate one terminates on the heart. The Lungs, *fe,* are very unequally lobed; for the left one is cleft into four lobes, while the right one is only bilobed. They are grouped round, and encircle, the heart, as the petals of a flower do the pistil in the centre. The Heart, *sin,* gives out from its basis (and here the air-tube joins it) three large vessels; the left one of which passes down along the spine to the kidneys, the central one goes to the liver, and the right to the spleen.

The office of the Diaphragm, *kih mo,* is to prevent (says the text) the rising up of unsavoury effluvia from the abdomen into the thorax. The stomach, *we,* is partially covered by the Omentum, *tche man,* and communicates with the Small Intestine, *seaou tchang,* and this terminates in the great gut *ta tchang,* at the ileo-cæcal valve, *lan mun*—where the separation between the solid and fluid parts of the intestinal contents takes place. While the latter find their way to the Bladder, the former are propelled forwards to the Rectum, *tche tchang.*

The Bladder, *pang konang,* opens outwardly by the Urethra, *neaou,* which receives in its course the seminiferous canal, *tsing.* The Spleen, *pe,* is made to communicate directly with the heart by a long canal, which passes directly through the diaphragm. The lobes of the Liver, *kan,* are represented encircling the gall-bladder, *lan,* in the same manner as the lungs are represented encircling the heart in the centre of their lobes. The Kidney, *chin,* is considered by the Celestial Anatomists as the essential organ of generation, charged with the fabrication of the semen: this is excreted by a long canal, which passes down along the spinal column, crosses the rectum, and terminates in the urethra. They also hold the opinion that the Heart is the seat or the principal organ, so to speak, of the sensitive Soul; while the Mind or intellectual being resides in the liver; and the Life, or vital essence, is seated in the centre of the chest.

The translation of the Chinese text, from which these particulars have been taken, was made by M. *Stanislaus Julien.*—*L'Experience.*

---

### M. LOMBARD'S REMARKS ON TYPHUS FEVER.

This excellent physician, having had the benefit of a British Medical Education, is exempt from one of the most besetting sins of most Continental writers—viz. a partial and exclusive view on the subject of Pyrexial pathology. He has recently contributed a series of elaborate papers to the French Medical Gazette on the Typhus or Typhoid Fever, which for a good many years past he has been in the habit of seeing in Geneva, where he resides. While he admits the great frequency of intestinal lesion in this malady, he does not fall into the egregious error of making this the "point de depart" of its etiology, or of supposing that its gravity is invariably commensurate with the amount of morbid change in the follicles and mucous glands of the bowels. He highly extols the judicious use of Calomel; and indeed seems to regard it—administered with discretion as a matter of course—as by far the most valuable remedy, in a great many cases. All that we propose to do, at the present moment, is to extract a few of the excellent remarks which the Doctor makes on the treatment of some of the most common complicating symptoms, which are apt to accompany this truly multiform, and often most perplexing of maladies.

*Intestinal Hæmorrhage.*—This should rarely, if ever, be regarded as a critical or salutary evacuation. It should therefore be checked without delay. One of the best remedies for the relief of this accident is unquestionably the acetate of Lead—in doses of one or two grains, with a quarter of a grain of extract of opium, every six or eight hours. Enemata with Goulard solution may also be administered. The extract of the Rhatany root, or of Logwood, and the decoction of the latter, will also be found very useful in many cases. Ice is one of the best things that the patient can take. He should remain very quiet and cool, and avoid everything that is likely to excite the bowels.

In the *Diarrhœa,* too, that is not unfrequently a most dangerous complication of Typhoid fever, the remedies now mentioned may generally be used with advantage. Sinapisms to the bowels also are often of great utility, when the relaxation is obstinate, and the debility of the system great: they should be kept applied, till considerable irritation of the skin is induced. The oxyde of Bismuth with Opium has succeeded in some inveterate cases. The Nitrate of Silver, and the Sulphate of Copper, have also been given with benefit.

In the *Pneumonia* and *Catarrh,* which not unfrequently complicate the course of Typhoid fever, the white oxyde of Antimony has been employed by us with almost uniformly good effects; it generally serves to allay the fever, to encourage perspiration, and also promote the expectoration of the sputa. In some cases, where the debility was very great, and there seemed to be a tendency to rapid exhaustion, we had recourse to the decoction of Polygala with subcarbonate of Ammonia, Musk, and Camphor; and witnessed, unquestionably, good effects from the treatment. When the Catarrhal Mucus was very abundant, and the expectoration difficult, the Muriate of Ammonia with Paregoric Elixir may often be given with benefit.*

The low *Delirium,* that is so frequently present in the progress and advanced

---

* This medicine—the Muriate of Ammonia, or Sal-ammoniac—is too much overlooked in the practice of British physicians. It has long been, and still remains, in high favour on the Continent, more especially among German practitioners. In a great number of cases of Bronchitis and Catarrh, it will be found a most excellent remedy, in combination with Squills or Antimony, and a little Henbane or Opium. Sir *G. Lefevre* has recently testified to its very useful effects in these and other diseases.—*Rev.*

stage of Typhoid fever, is best combated by the use of Camphor and Opium, along with an occasional blister to the neck. This latter remedy will generally succeed in removing that intense headache which not unfrequently afflicts Typhus patients, during the convalescent stage.

*Anasarca* and *Ascites* are occasional consequences of fever. One of the best remedies is the Chlorate of Potash, in doses of 15 or 18 grains every four or six hours. Covering the dropsical limbs with oiled silk has seemed to promote the good effects of internal medication. We have rarely used Digitalis or other diuretics, as the chlorate has generally proved quite sufficient for the cure of the disease; it has this great advantage over most remedies, that it generally improves, rather than impairs, the digestive function.

*Salivation* is apt to be a troublesome consequence of the administration of mercury in fever, at least in certain constitutions. No remedy has in our hands proved more useful against this distressing accident than the application of two or three leeches under the lower maxilla: the symptoms usually subside rapidly and effectually under this simple treatment. A gargle made with alum, or camphorated spirits of wine, will also be found very useful in many cases.* Mild saline aperients at the same time may be given with advantage.

---

### Philosophical Anatomy.

M. *Serres* remarks that, " a general law of Nature is, that the various organisms become more and more decomposed and subdivided (fractionnées), in proportion as we descend in the animal scale from Man to the lower Invertebrata. By this fractionary division, the complexity of certain organisms—which in Man and the higher Vertebrate animals are often so inextricable—becomes more and more simplified, so that, in the creatures at the bottom of the zoological scale, we find them reduced to their most elementary configuration. Another Law, of not less general application, is that, if we follow the development of a complex organism —through its successive stages or phases of evolution—we find that it appears first in a state of remarkable simplicity; and that each successive transformation, which it undergoes, renders it more and more complex, until it arrives at its perfect and matured development.

But this is not all: for, if we compare the organology of the lower and more simple animals with the primary development of the higher Vertebrata, we discover numerous examples of a marvellous analogy.

All the various methods, which anatomists employ to unravel the intimate texture of any organ, serve only to bring it back to that more simple condition, in which it existed at an early period of its development. Whether we use the scalpel, or trust to maceration, or erosion with acids, &c., these means act only by destroying the cementing medium or bond of union, which holds the constituent parts together in one whole."

---

### Analogy between some Monstrosities and the Lower Animal Forms.

" In several respects, there is no inconsiderable analogy between the normal and mature configuration of many of the Invertebrate animals and the structural

---

* A blister to the throat and a gargle of brandy and water are *the* remedies for troublesome salivation.—*Rev.*

Anomalies, or—as they are usually called, Monstrosities—of the Vertebrata. Thus, several of the Polypes are *anenteric,* i. e. destitute of an intestinal canal; so are the Moles, which are not unfrequently discharged from the uterus. In other cases, we find the anterior part only of the alimentary tube to be present: a similar configuration has been often met with in imperfectly-formed embryotic productions. It is well known that some monsters are Acephalous, while others are entirely destitute of a heart. We need scarcely say that the Analogues of these conditions are frequent in the members of the lowest animal groupes. Such privations of organs are indeed incompatible with the extra-uterine life of Vertebrate animals; but let it not be forgotten, that the imperfectly-formed Being was alive while in the womb of the mother; it had passed through a certain stage of existence, and had already completed its life as an invertebrate animal."

---

### THE PROCESS OF SECRETION.

The following extract from Dr. *Mandl's* recently published "Manual of General Anatomy applied to Physiology and Pathology," deserves notice, as bearing on the important Doctrine of Cell-formation. "The greater number of the fluids, which constitute the basis of the different secretions—such as the gastric and intestinal juices, the saliva, tears, milk, mucus, wax of the ears, fat, &c.—proceed from a gradual dissolution of the substance of the very glands, which are generally supposed to eliminate them. The Blood, no doubt, furnishes certain elements for each secreted fluid; but that, which constitutes the characteristic constituent of each secretion, is the fluid contained in the microscopic *cells,* which enter into the formation of every gland:—this fluid is poured out in consequence of either the bursting, or the dissolution, of the cellular envelopes. The *Cells,* which along with the *blastema* constitute the parenchymatous substance of glands, are developed within the minute secreting canaliculi. When they have attained to a certain degree of maturity, they detach themselves from the interior, and are carried along in the secreted fluid."

---

### FRACTURE OF THE BASE OF THE SKULL FROM FALLING ON THE FEET.

The following very interesting case recently occurred in the practice of M. *Robert* at the Hospital Beaujon. A middle-aged robust man fell from a height of 35 feet, and alighted direct upon his feet: this was on the 24th of May. He was stunned a little at the time, but experienced no other inconvenience. Next day he resumed his work as usual. On the fourth day, he complained of a severe pain in the right ear, and of not being able to sleep. After the lapse of three weeks from the date of the accident, a very intense headache came on; and then, for the first time, the right eye was observed to be turned inwards. He entered La Pitié hospital, where he was treated by M. *Nonat* with venæsection, leeches, blisters and morphia. On leaving this hospital, he entered the Beaujon hospital. The severe headache still continued; but there was no symptom of paralysis either of the motory or sensory functions. He remained nearly in the same state —without either aggravation or amendment of the symptoms—until the 20th of September, when he was seized with violent Delirium. He was immediately bled and leeched, but without avail; for he died within 24 hours after the attack— three weeks after his admission into the Beaujon hospital, and nearly four months after the occurrence of the accident. On *Dissection,* the two clinoid processes were found separated from the body of the sphenoid bone. The petrous portion of the right temporal bone also was fractured across, and a tolerably-sized piece fairly detached. The Brain and its envelopes appeared to be little, if at all, affected; but the sixth pair of nerves was lacerated at the seat of the fracture. —*L'Experience.*

### Case of Elephantiasis, or Barbadoes Leg.

M. *Cazenave* relates a case of this disease, which occurred in a middle-aged woman, whose general health appeared to be quite sound, but who had suffered two or three attacks of Erysipelatous inflammation in the right (the affected) lower extremity. The limb had acquired an immense size, when she was admitted into St. Louis Hospital; "the leg and thigh, confounded together, resembled a huge shapeless pillar, without any distinction of parts." The skin was uniformly hard and resistant; but it did not present to the touch any traces of indurated cords or ganglia. The patient complained of no pain or tenderness, but only of a sensation of unpleasant coldness in the part; and she found great difficulty in keeping it warm. M. *Cazenave* directed her to take a strong decoction of Guaiacum and Mezereon; to use vapour baths; to rub the leg with the Ung. hyd. potassæ; and to have it bandaged from the toes upwards: absolute rest also in bed was enjoined. This mode of treatment was persevered in from the 9th of August to the 28th of November, at which date the patient was discharged almost completely cured.

What is the proper pathological character of such a case as this? M. *Cazenave* says that "it is a simultaneous or successive affection of the skin, the cutaneous lymphatic vessels and glands." This is certainly not a very satisfactory explanation. Is it not rather an infiltration of a viscid serum into the subcutaneous Cellular tissue, producing distention, and ultimately thickening and induration, of the skin? Whether the primary lesion be an affection of the lymphatic vessels or not, we cannot as yet decide; but it is abundantly obvious that an analogy may be traced between this kind of Elephantiasis and the Phlegmasia alba dolens of puerperal women.

(The practice, adopted in this case, appears to have been extremely judicious. The Guaiacum and Mezereon often exert a very marked influence on the cutaneous circulation, stimulating the exhalants, and invigorating the capillary vessels: hence they are excellent remedies in many cases of chronic skin-disease, rheumatism, &c.)

### Leprosy in Sardinia.

From an official report on this subject, recently published by order of the Sardinian Government, we learn that there are, at the present time, upwards of 100 cases of genuine Leprosy in the different provinces of the kingdom. The disease is said to be decidedly contagious, at least in its advanced or suppurative stage—when it seems to be communicable not only by contact with a diseased individual, but also by his wearing-apparel. Cases of this loathsome malady are occasionally met with in the Southern provinces of France also, as in some parts of Provence, in the neighbourhood of Marseilles, &c. Dr. *Trompeo*, who was employed by his government to report upon the subject, states it as his opinion that the Leprosy, as seen in Sardinia at the present time, is essentially the ancient disease of the Egyptians and Greeks, modified indeed somewhat by lapse of time and the influence of modern civilisation. In a few cases, the patients exhibited each and every one of the symptoms and characters of the genuine Leprosy, such as we read of in the authors of antiquity.

### THE VALERIANATE OF ZINC—A NEW ANTISPASMODIC.

"Since the beautiful researches of Prince *Louis Buonaparte* on the preparations of Valerianic Acid, a most useful and potent remedy for a variety of nervous affections has been introduced into medical practice. The Valerianate of Zinc is easily formed by adding the protoxide of the metal to the vegetable acid, until this be saturated: the salt is obtained by slowly evaporating the solution. The dose is one or two grains at a time, taken in the form of a pill, It has been used with very decided advantage in various forms of Neuralgia by several Italian physicians, as we find recorded in a late Number of the *Bulletino delle Scienze Mediche*.

(Supposing that this new salt be really useful in the cases alluded to, is it likely to have the same efficient virtues as the Ammoniated Tincture of Valerian—a truly excellent anti-neuralgic—with the white Oxyde or the Sulphate of Zinc? We should think not.)

---

### INOCULATION OF VERATRIA IN NEURALGIA.

M. *Lafargue* has, in many cases of Prosopalgia, had recourse to this endermic mode of treatment with singularly good effects. The plan he follows is nearly the same as that used for Vaccination: a number of punctures are made with the point of lancet, that has been charged with a saturated solution of the Alkaloid. Each puncture becomes at once the seat of a sharp pain, which is usually compared by the patient to a continual deep pricking with the point of a needle. This unpleasant sensation lasts from five to fifteen minutes, and then gradually subsides; and, with it, the red areola that has formed around the punctured spot. M. *Lafargue* recommends, in severe cases, that the inoculation be repeated morning and evening; and that as many as ten or twelve punctures should be made at a time. He has used the same method of treatment with decided good effects, in several cases of partial Paralysis. (This simple and elegant mode of using Veratria might possibly be of considerable service in some cases of Amaurosis.)

---

### NUX VOMICA IN NEURALGIA.

M. *Roclants*, a Dutch physician, reports most favourably of the effects of this potent drug in severe cases of Neuralgia of the face and other parts, and communicates at the same time the therapeutic results obtained by many of his professional friends. Out of 29 severe cases, a perfect cure was effected in 25, and decided relief was afforded in the other four. The dose, in which the powdered Nux Vomica was administered, was from three to ten grains, and upwards, in the course of the twenty-four hours. In all cases its effects should be narrowly watched, as unpleasant consequences have occasionally resulted from incaution on the part of the physician. M. *Roclants* is inclined to regard the Nux Vomica as, on the whole, the most efficient and certain remedy against severe Neuralgia; he has seen several cases, which had resisted the prolonged administration of steel, bark, and all the other most approved means, yield to its use.

M. *Trousseau* has recently been very strongly recommending the Strychnos as a most valuable remedy in obstinate Chorea.

---

### Utility of Musk in certain Cases of Delirium.

It is M. *Recamier*, we believe, who has most strongly advocated the use of this powerful antispasmodic in certain forms of Delirium, occurring in the course of various febrile and inflammatory diseases. When Pneumonia, as in certain constitutions, and in certain epidemics, is accompanied with marked symptoms of cerebral disturbance,—a very embarrassing complication—the use of Musk, either alone or in combination with Calomel, has been often found to be of decided advantage. It is also very useful in the delirium which not unfrequently attends the course of Erysipelas, and several other exanthemata; more especially in small-pox, during the maturation and desiccation of the eruption.

Dr. *Roche* of Strasbourg has recently published several very instructive cases in illustration of this practical point. The first was one of Erysipelas of the face and head; the second of gangrenous Sore throat; the third of Scarlatina; and the fourth of Variola. In all these, the Musk appears to have acted very beneficially. He adds: "I have employed it also in two cases of furious Mania; the violent agitation was arrested; but no other good was produced. It completely failed in a case of grave Typhoid fever, and also in one of acute Bronchitis, accompanied with delirium, which occurred in a middle-aged man of very intemperate habits." He closes his observations with the following general remark: "It appears to me that the administration of Musk is indicated whenever, in the course of acute diseases, Delirium supervenes without any distinctly appreciable cause, and the severity of which is not commensurate with that of the primary disease. In very many cases, I have had the satisfaction of witnessing cures, which I could not certainly have anticipated before my acquaintance with this most valuable remedy. The first effect, which it usually produces when successful, is to induce a quiet refreshing sleep, and a general tranquillizing influence over the entire body: sometimes it induces slight nervous twitchings in the eyelids, the extremities &c."—*Journal des Connaiss. Med. Chir.*

*Remarks.*—Musk is unquestionably one of the most potent, and least fallible, antispasmodics that the Pharmacopœia contains: the only drawback to its general use is its expense. Fortunately Assafœtida, Galbanum, and good Castoreum may very generally be substituted for their more costly analogue. In all cases of nervous agitation, unconnected with plethoric and inflammatory excitement, this class of antispasmodic medicines may be used with advantage. Camphor also is a very potent member of the same family; and few compounds are more beneficial than pills composed of musk or assafœtida and camphor,—to which a few grains of Calomel, and also some extract of Henbane, may often be most judiciously added. We have witnessed most pleasing effects from this formula in several cases of puerperal Mania.—*Rev.*

---

### M. Velpeau on the Use of the Nitrate of Silver in Opthalmia.

The following is a summary of this ever active surgeon's opinions on an important point of practice.

1. The Nitrate of Silver is unquestionably the best local application that can be used in a great many diseases, acute as well as chronic, of the Eye.

2. In various kinds of Blepharitis, the nitrate is most advantageously used in the form of pommade.

3. In inflammation of the lids, the direct application of the solid caustic is usually attended with the greatest benefit.

4. For the slighter forms of Conjunctivitis, a weak solution of the salt in distilled water is best. In the purulent form of the disease, the solution must be used considerably stronger; but the solid nitrate is not unfrequently found to suit better in such cases.

5. In the treatment of the different forms of Ophthalmic inflammation, it will often be found of great utility alternately to increase and diminish the strength of the application—whether this be in the form of ointment or of solution.

---

### TARTRATE OF ANTIMONY IN INJURIES AND OPHTHALMIA.

Professor *Lallemand* of Montpellier has long been in the habit of using the tartrate, administered in large doses, in numerous cases of wounds, traumatic lesions, inflammation of the eye, joints, &c. when the object is to subdue inflammatory excitement, without permanently impairing the strength of the patient. This is often the aim and desire of the surgeon, in hospital practice more especially; and indeed whenever he has to do with persons whose constitutions may have been enfeebled either by insufficient diet, or by intemperate habits. Several cases of severe Purulent Ophthalmia are related, wherein the antimonial treatment was attended with the most pleasing results, even after the use of mercury had been pushed to a great extent, but without avail. The Professor recommends the application of blisters to the nape of the neck at the same time. In this way a powerful revulsion from the affected organ is established, while the activity of the general circulation is subdued.

---

### M. GERDY ON CONGENITAL LUXATIONS.

" These deformities are attributable to a malformation, or abnormal condition, of articular development, which either prevents the bones from being articulated with each other at all, or which favours their displacement, when the articulation really exists. In some cases, therefore, there can be no proper luxation, because there is no articulation; while, in other cases, the luxation is apt to occur—either before or after the period of birth—from the very slightest causes, in consequence of the imperfect development of the articular mechanism. When the malformation affects the hip-joint, we usually find, on dissection of the parts, that the cotyloid cavity is either too wide or too narrow: or that the head of the femur is excessively large or diminutive; or that its cervix has a faulty direction, or has become agglutinated to the coxal bone; or that the round ligament is absent, divided, or excessively elongated; or that there is some deviation of the pelvic bones, or an abnormal configuration of their structure; or, lastly, that there is some lesion of the vertebral column."

---

### ON THE PARALYTIC AFFECTIONS OF INFANCY.

M. *Serres* remarks, that the Paralytic Affections, which are occasionally met with in infants and young children, are often owing to an arrest in the normal development of the parts affected; or to a want of harmony between the development of the bones and that of the muscles; or to an over-rapid growth and increase of one section—as, for example, of the upper half—of the body, compared with the growth and increase of another. The febrile diseases of infancy and childhood not unfrequently leave behind them a loss of motion, and sometimes of sensation also, in one, or more than one, limb.* The convulsions, which so often arise from the presence of worms in the bowels as well as from other morbid states in early life, may be followed by a similar affection of the muscular and nervous systems. After the exciting causes have been once removed, the use of Strychnine, externally as well as internally, has been adopted with advantage. Cold bathing and the exhibition of steel are also very serviceable in many cases.

### Of the Cause of Rabies in the Dog.

It is no new idea that this frightful malady is attributable, in the case of the dog, to the forced deprivation of sexual intercourse. But—as M. *Taffoli* (in one of the numbers of the Annali Universali di Medicina, for last year,) observes,—however just this hypothesis may be in principle, it is obvious that it must not be insisted upon too peremptorily or unreservedly; for we well know that a multitude of dogs are continually in a state of unnatural celibacy, and yet the disease in question is far from being of very common occurrence. We may therefore very reasonably suppose, with this gentleman, that the animal must be previously predisposed, somehow or other, to its development. "Observe," says he, "around a bitch in *heat*, the dog that is the most excited; it is usually an animal of *sagacity* and *sentiment*, and of a quick and choleric temper. If this animal be thwarted and prevented from indulging the sexual desire, we may almost with confidence predict that it will be in him, if in any, that Rabies will make its appearance!"

### Nævi treated by Inoculation with Croton Oil.

A correspondent of the French Academy recommends, that five or six punctures be made, on the surface and around the circumference of *Nævi Materni,* with a lancet charged with Croton Oil—used, therefore, in the same manner as the vaccine lymph has been recommended for the same purpose. Each of the punctures becomes the seat of a small boil. From the coalescence of the several furuncular pustules, the whole of the erectile tissue is affected in the first instance with an inflammatory, and, subsequently, with a suppurative and ulcerative action. The writer does not mention whether he has ever realised his idea, or put it to the test of actual experience.

### Chloruretted Lotions in Confluent Variola.

Dr. *Bailleul* strongly recommends the application of chloruretted lotions to the skin, throat and nasal passages, in severe cases of confluent Small-pox. He is

* Sir *Walter Scott's* case seems to have been of this kind. In his auto-biographical sketch, he says:—" I shewed every sign of health and strength, until I was about 18 months old. One night I shewed great reluctance to be caught and put to bed, and, after being chased about the room, was apprehended and consigned to my dormitory with some difficulty. It was the last time that I was to shew such personal agility. In the morning I was discovered to be affected with the fever, which often accompanies the cutting of large teeth. It held me three days. On the fourth, when they went to bathe me as usual, they discovered that I had lost the power of my right leg."

of opinion that they serve to decompose the purulent matter in the pustules, and thereby to counteract the tendency that often exists to cutaneous and pulmonary Asphyxia, by neutralizing the noxious agency of the purulent matter on the cutaneous and mucous surfaces. The fever of resorption—or Secondary Fever, as it is usually called—is thus rendered much less formidable; and, moreover, the emanation of poisonous effluvia from the body is in a great measure arrested. Considered as local applications, the chloruretted lotions refresh and re-invigorate the vital properties of the skin, and tend also to promote the cicatrisation of the greyish-coloured ulcers which line the bottom of the variolous pustules. The practice here recommended may be expected to be of especial utility in prisons, hospitals, on board ship, &c., where patients are apt to be crowded together, and the danger of infection is therefore to be most dreaded.

---

### GERMAN HYDROPATHIC JOURNAL.

We have received three Numbers of the "Neue Wasserfreund" (New Waterfriend) edited by Dr. *Schmitz,* the superintendent of a sluicing establishment at Marienberg. As a matter of course, they are almost entirely taken up with long lists of cures and recoveries effected by the "Priessnitzschen Heilweise," and are therefore not worthy of any notice from us; the object of the contributors being mainly to advertise their success. There is a brief notice, in one of the Numbers, of some English pamphlets and books on the subject, and, among others, of Sir *Charles Scudamore's* "Visit to Graefenberg." Dr. *Schmitz* is a politic man; he lauds the Knight as one of the most distinguished physicians of the British metropolis, and extracts a laudatory notice of himself from the credulous Knight's good natured pages! He then, as a finale, gives a list of the cures performed at his establishment during the preceding nine months: among numerous Kaufmanns, Rentners, Herrs, Fraus and Frauleins, figures conspicuously Herr *Mayo,* professor of Surgery and Operator from London! Is not the ex-surgeon of the Middlesex Hospital an attaché, or perhaps a part proprietor of Dr. *Schmitz's* establishment? We surely saw a notice to this effect in some recent periodical.

---

### URETHRAL PAINS RELIEVED BY COMPRESSION.

M. *Vidal,* struck with the well-known fact that patients often succeed in relieving themselves of severe pain in the urethra by firmly compressing the penis, thought it probable that, by employing a more uniform and prolonged pressure, greater benefit might be obtained. With this view, he recommends that the penis be encircled with numerous strips of adhesive plaster; and assures us that he has witnessed excellent effects from this simple plan. (In the course, and towards the decline, of Urethritis, there is often experienced a most distressing pain about the neck of the bladder, immediately before and after the expulsion of the urine: it appears to arise from a spasmodic contraction of the sphincter and the detrusor muscles. By far the most decided and speedy means of relief is firm compression, with the hand, of the perineum in front of the anus.)

---

### ASPHYXIA INDUCED BY AN EXCESSIVE QUANTITY OF THE AMNIOTIC FLUID.

Dr. *Levrat* of Lyons reports, in his Recherches Medico-Chirurgicales recently published, five cases of Asphyxia in pregnant women, which seemed to him to be induced by an excessive quantity of intra-uterine fluid. In three of the cases, the symptoms were relieved by puncturing the membranes with the finger, and thus giving issue to the superabundant quantity of amniotic water. In one case, a trocar was introduced, as in the ordinary operation of paracentesis, at the usual place, and a similar result was obtained. In the last case the patient died, before relief was procured. On opening the uterus after death, it was found to contain upwards of thirty pints of fluid. In all these cases, the women had reached nearly the full term of pregnancy.

---

### Pommade for Chaps and Fissures of the Toes.

One of the most annoying effects of secondary Syphilis is the formation of fissures on the internal surface of the toes: they are usually very painful, are surrounded with a red areola, and secrete a syphilitic matter. In a few cases, gangrene has been known to supervene, and to destroy one toe after another. An ointment, containing litharge, white precipitate, and a few drops of laudanum, has been used with very marked success in such cases, in many of the hospitals of Germany. It is, also, much recommended in the serpiginous and phagedænic ulcers, which occasionally supervene upon vaccination in children of a scrofulous or syphilitic constitution. The process of cicatrisation is often promoted by bathing the sores, at the same time, with a decoction of hemlock and marsh-mallows.

---

### Pharmaceutical Formulæ.

1. *Anodyne Pommade.*

| | | |
|---|---|---|
| Take of, | Galen's Cerate . . . . . . . . . . . . | 31 parts, |
| | Extract of Belladonna . . . . . . . . | 8 —— |
| | Acetate of Morphia (previously dissolved) . | 3 —— |

Mix well together.

This Pommade is exceedingly useful in cases of muscular pains, chronic rheumatism, &c. when rubbed on the affected parts.

2. *Ustiocure Cream.*

The following preparation has produced, in my practice, 'de si beaux, de si constants succes' in cases of Burns, that I have great confidence in recommending it to my medical brethren.

Take of, Chloruret of Lime,
The White Oil of Commerce,
of each, Equal parts,

Blend them well together, to make a smooth ointment. It should be applied on fine linen, or (what is better) on taffetas that is 'gommé et fenetré.'

3. *Lozenges, &c. of Copaiba.*

Take 30 parts of Copaiba Balsam and 12 parts of Calcined Magnesia; mix them well together, and set the mass aside for 24 hours; then divide it into portions of a suitable size, and roll them into proper shape between the fingers. They are then to be put into a tin basin, and, after being sprinkled first with gum water and then with powdered sugar, to be well shaken about for some time. When sufficiently crusted over, they are to be placed upon a hair-sieve, and dried in a stove.

4. *Anti-scrofulous Syrup.*

| | | |
|---|---|---|
| Take of, | Cod Liver Oil. . . . . . . . . . . | 125 parts. |
| | Extract of Walnut-leaves . . . . . . | 45 —— |
| | Honey . . . . . . . . . . . . . . . | 735 —— |
| | Ioduret of Potassium . . . . . . . | 6 —— |
| | Tincture of Quinine . . . . . . . | 6 —— |
| | Syrup . . . . . . . . . . . . . . . | 1000 —— |
| | Essence of Aniseed . . . . . . . . | 240 —— |

Mix slowly and well together.

The nauseous taste of the cod-oil is disguised, while its anti-scrofulous properties are aided by the other substances combined with it.

---

### ON THE PROPER AGE FOR FEMALES TO MARRY.

M. *Raciborski,* in his recent elaborate Memoirs on this subject, makes the following curious observations :

"M. *Marc* says—and we think that he is quite right in the statement—that the strength and the vigour of the offspring are more dependent upon the state of the mother's than of the father's constitution. The eggs, for example, of very young hens are always small, however lusty be the cock that has fecundated them. The same holds good in the case of Calves, Colts, &c.

According to the tables in the late Mr. Sadler's work, the average offspring of each marriage in England, when the mother is below 16 years of age, is 4,40; when her age is from 16 to 20, it is 4,63; when from 20 to 23, it is 5,21; and when from 24 to 27, it is 5,43. If these calculations be correct, they afford the most convincing evidence that, not only the number, but also the strength and *viability,* of children born, are much influenced by the age of the mother. According to our views, the interval between the 20th and the 24th years is the most advisable for marriages among the women of this country (France). The testimony of an accomplished female writer on the education of young persons may be aptly quoted here. "We are in the habit of marrying our daughters so young," says *Madame de Remusat,* "that they really have not had the time to look at or understand anything in its proper light. If established usages could be suddenly broken through, and if we were to consult Nature more in our matrimonial arrangements, I believe that the age of 25 years, or so, is that which would be considered most advisable for young women to marry. But, alas! there is little hope that Fashion will recognise so great a change in her customs as this. We should at least wait till a girl has passed her 20th year; and meanwhile everything should be done, by a judicious system of education, to hasten on the maturity of her reason."

